# Functional genomics analyses of RNA-binding proteins reveal the splicing regulator SNRPB as an oncogenic candidate in glioblastoma

**DOI:** 10.1186/s13059-016-0990-4

**Published:** 2016-06-10

**Authors:** Bruna R. Correa, Patricia Rosa de Araujo, Mei Qiao, Suzanne C. Burns, Chen Chen, Richard Schlegel, Seema Agarwal, Pedro A. F. Galante, Luiz O. F. Penalva

**Affiliations:** Centro de Oncologia Molecular, Hospital Sírio-Libanês, São Paulo, Brazil; Children’s Cancer Research Institute, UTHSCSA, San Antonio, TX USA; Georgetown University Medical Center, Washington, DC USA; Department of Cellular and Structural Biology, UTHSCSA, San Antonio, TX USA

**Keywords:** RNA-binding proteins, Glioblastoma, Glioma stem cells, SNRPB, Splicing

## Abstract

**Background:**

Glioblastoma (GBM) is the most common and aggressive type of brain tumor. Currently, GBM has an extremely poor outcome and there is no effective treatment. In this context, genomic and transcriptomic analyses have become important tools to identify new avenues for therapies. RNA-binding proteins (RBPs) are master regulators of co- and post-transcriptional events; however, their role in GBM remains poorly understood. To further our knowledge of novel regulatory pathways that could contribute to gliomagenesis, we have conducted a systematic study of RBPs in GBM.

**Results:**

By measuring expression levels of 1542 human RBPs in GBM samples and glioma stem cell samples, we identified 58 consistently upregulated RBPs. Survival analysis revealed that increased expression of 21 RBPs was also associated with a poor prognosis. To assess the functional impact of those RBPs, we modulated their expression in GBM cell lines and performed viability, proliferation, and apoptosis assays. Combined results revealed a prominent oncogenic candidate, *SNRPB*, which encodes core spliceosome machinery components. To reveal the impact of SNRPB on splicing and gene expression, we performed its knockdown in a GBM cell line followed by RNA sequencing. We found that the affected genes were involved in RNA processing, DNA repair, and chromatin remodeling. Additionally, genes and pathways already associated with gliomagenesis, as well as a set of general cancer genes, also presented with splicing and expression alterations.

**Conclusions:**

Our study provides new insights into how RBPs, and specifically SNRPB, regulate gene expression and directly impact GBM development.

**Electronic supplementary material:**

The online version of this article (doi:10.1186/s13059-016-0990-4) contains supplementary material, which is available to authorized users.

## Background

Glioblastoma (GBM) is the most common and lethal tumor type of the central nervous system, with 16,000 new cases per year in the US alone [1]. GBM is highly heterogenic, invasive, and refractory to the current standard of care, which is a combination of surgical resection, adjuvant radiotherapy, and temozolomide [2]. Despite decades of research, the overall outcome for patients with GBM remains extremely poor, with an average survival of approximately 15 months after diagnosis [1, 3–5].

To identify new targets for therapy, The Cancer Genome Atlas (TCGA) consortium produced a comprehensive somatic landscape of GBM through a set of genomic, epigenomic, transcriptomic, and proteomic analyses, combining molecular and clinical data for 543 patients [6, 7]. These analyses have improved our understanding of GBM pathobiology, emphasizing that gliomagenesis is driven by signaling networks with functional redundancy, which allows adaptation in response to therapy. Because novel therapeutic strategies based on these findings have not yet become a reality, it is necessary to investigate additional pathways of gene deregulation in GBM. Equally important is the study of glioma stem cells (GSCs), which are particularly relevant to tumor initiation and resistance to treatment [8–10]. Unveiling individual genes and pathways that contribute to GSC survival and phenotype maintenance might enable the design of novel therapeutic strategies against GBM.

RNA-binding proteins (RBPs) are master regulators of co- and post-transcriptional mechanisms, including RNA processing (splicing, capping, and polyadenylation), transport, decay, localization, and translation. They are still a poorly characterized class of regulators, with hundreds of new members only recently identified via novel experimental high-throughput approaches [11–13]. The most updated human RBP catalog includes 1542 genes [14], which represents ~7.5 % of human coding genes (GENCODE version 19 [15]). Mutations and alterations in RBP expression levels, which have been observed in many tumor tissues [16–18], are known to impact large gene sets and to contribute to tumor initiation and growth. In fact, numerous well-characterized RBPs such as HuR, Musashi1, Sam68, and eIF4E have been implicated in multiple tumor types [19–22]. In the context of neural tissue, the number of tissue-specific RBPs and alternative splicing isoforms is particularly high compared with other tissues [14, 23–25]. Hence, RBPs play key roles in this biological context and their alteration is expected to be a major contributor to gliomagenesis. Some important players include Musashi1 [26–28], HuR [27], hnRNP proteins (H and A2/B1) [29–32], and PTB [29, 33, 34].

In order to identify novel RBPs potentially implicated in GBM development, we conducted a combination of transcriptomic analyses followed by functional screenings. We found 58 genes with oncogenic potential, defined as those with high expression in GBM and GSC samples relative to their normal counterparts. Twenty-one of these genes are also associated with a poor prognosis and were further selected for functional analyses. *SNRPB*, which encodes core components of the spliceosome complex SmB/B’, showed the strongest impact on viability, proliferation, and apoptosis. We determined that changes in SNRPB expression levels have a large impact on expression and splicing regulation and preferentially affect alternative exons and introns. RNA processing, DNA repair, and chromatin remodeling are among the biological processes with the highest number of genes affected by SNRPB at expression and splicing levels. Moreover, several genes in pathways relevant to GBM initiation and development, such as *RTK*, *PI3K*, *RAS*, *MAPK*, *AKT*, *RB*, and *p53*, as well as a set of additional cancer genes, displayed alterations in their splicing and expression profiles upon *SNRPB* knockdown.

## Results

### Several RBPs are aberrantly expressed in GBM and GSCs

**Fig. 1:**
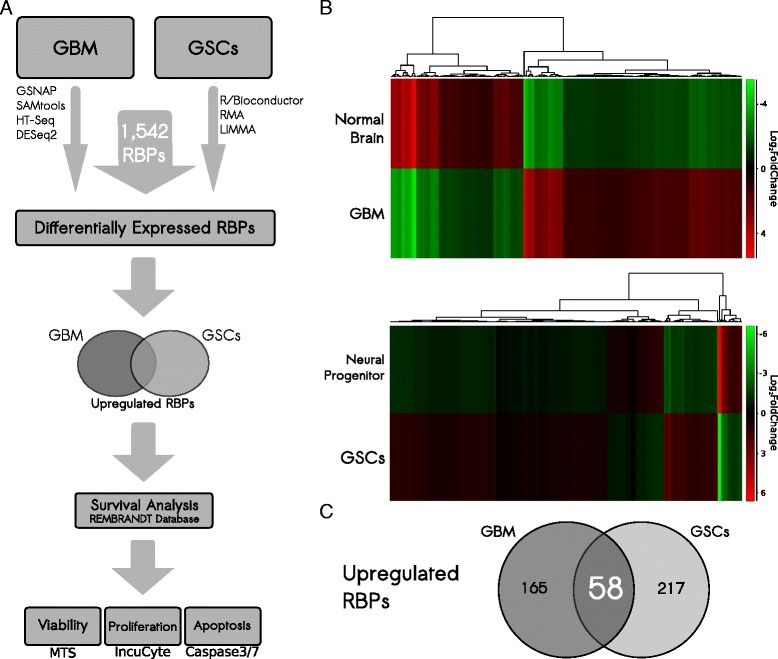
Experimental design and RNA-binding protein (*RBP*) expression profile in glioblastoma (*GBM*) and glioma stem cells (*GSCs*). **a** Gene expression results from GBM and GSC samples were combined to identify upregulated RBPs. Those RBPs were evaluated regarding their association with survival decrease, and their functional impact in GBM was assayed by a set of functional assays. **b** Heatmaps show all differentially expressed RBPs in GBM samples from The Cancer Genome Atlas compared to normal brain samples and in GSC samples compared to a normal neural progenitor cell line. **c** Venn diagram shows the intersection between gene expression analyses

To identify RBPs potentially involved in GBM development, we examined the expression profiles of all 1542 human catalogued RBP coding genes [14] in two different contexts: GBM samples from TCGA versus normal brain; and GSCs versus normal neural progenitor cells (Fig. 1a). We obtained raw RNA sequencing (RNA-Seq) data for 170 GBM samples from TCGA database (Additional file 1: Table S1) and compared them with 14 normal brain samples: eight samples from two studies available in the Sequence Read Archive (SRA), one sample from the Human Body Map, and five samples from TCGA (see ‘Methods’; Additional file 1: Table S1). This approach allowed the identification of 223 upregulated and 135 downregulated RBPs in tumors compared to normal samples (Fig. 1b top panel; Additional file 1: Table S2). Next, we looked at the expression of these differentially expressed RBPs, classifying all samples according to the four molecular GBM subtypes (classical, neural, proneural, and mesenchymal) to identify particular associations (if any). Results indicated that the overall expression profile was very similar among subtypes, with no differentially expressed RBPs showing drastic changes across subtypes (Additional file 2: Figure S1).

GSCs constitute a unique subpopulation within the tumor and display features similar to normal stem cells [35]. Their association with tumor relapse is often linked to their tumor-initiating capacity as well as radio- and chemoresistance [35–38]. Therefore, identifying regulators that maintain GSC phenotypes and/or contribute to their survival is critical for designing novel therapeutic strategies. We examined the microarray dataset of Mao et al. [39] to identify differentially expressed RBPs in GSCs in comparison to normal neural progenitor cells. This analysis revealed a total of 275 upregulated and 85 downregulated RBPs in GSCs (Fig. 1b bottom panel; Additional file 1: Table S3).

We focused next on the identification of “pro-oncogenic RBPs.” We selected these RBPs because they tend to be more attractive targets in therapeutic contexts [40] and they are easier to handle in screening studies [41]. Results from both transcriptomic studies were merged: 58 genes were determined to be upregulated in both GBM and GSC samples (Fig. 1c), which represents a highly significant overlap (*p*-value = 0.0006; hypergeometric test). Those 58 genes were selected for further analyses.

### Upregulation of RBPs is associated with decreased survival and is prevalent in higher grade gliomas

**Fig. 2:**
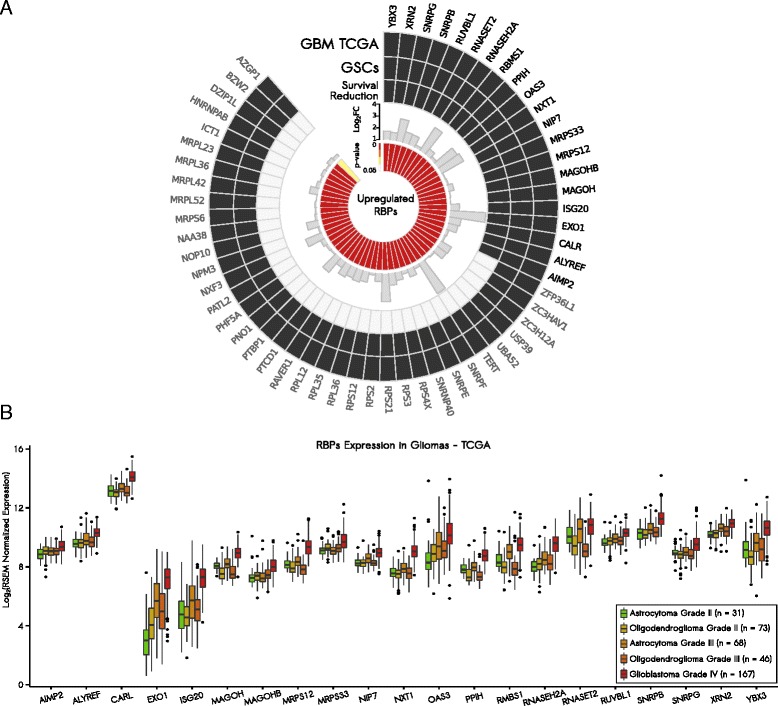
Candidates’ selection and characterization. **a** Circos plot shows 58 upregulated RNA-binding proteins (*RBPs*) in glioblastoma (*GBM*) samples from The Cancer Genome Atlas (*TCGA*) and glioma stem cell (*GSC*) lines. Fold-changes and corrected *p*-values were extracted from the RNA-Seq analysis (*GBM TCGA*). Twenty-one RBPs were also associated with survival reduction and were further investigated. **b** RBPs exhibited higher expression levels in GBMs compared with lower grade gliomas

To determine whether our set of 58 pro-oncogenic RBPs exhibits an association with poor prognosis in gliomas, we used clinical and expression data from the REMBRANDT database [42]. We built Kaplan-Meier survival curves comparing samples with increased expression of the selected RBPs to all other samples. Twenty-one out of the 58 upregulated RBPs showed an association with survival reduction when overexpressed (*p*-value < 0.05; log-rank test; Additional file 2: Figure S2). Figure 2a presents a summary of the selected RBPs and their results in survival analysis.

We also evaluated gene expression levels of these RBPs using a large cohort of normal brain samples generated by the Genotype-Tissue Expression (GTEx) Project [43]. By comparing expression levels of the 21 RBPs in 222 normal brain samples from GTEx with 170 GBM samples from TCGA, we were able to confirm the overexpression of our selected RBPs in GBM samples (Additional file 1: Table S4).

Finally, to corroborate the relevance of these 21 selected RBPs in an additional context, we evaluated their expression levels in 167 GBM samples (grade IV glioma) versus 218 lower grade glioma samples (grades II and III astrocytomas and oligodendrogliomas) from TCGA. In general, analyzed RBPs exhibited higher expression levels in GBM samples than in lower grade glioma samples (*p*-value < 0.001; Wilcoxon rank-sum test; Fig. 2b; Additional file 1: Table S5). The only exception was RNASET2, which presented no significant difference in one comparison (*p*-value = 0.1428 for GBMs versus grade III astrocytomas; Wilcoxon rank-sum test; Fig. 2b; Additional file 1: Table S5).

### Analysis of regulatory elements potentially triggering overexpression of RBPs in GBM

To try to identify mechanisms responsible for the upregulation of RBPs in tumor samples, we evaluated whether the 21 selected RBPs are targeted by frequently downregulated miRNAs in GBM (tumor suppressor miRNAs). Using a list of tumor suppressor miRNAs compiled by Hermansen and Kristensen [44], we found that 18 of those miRNAs potentially target 15 out of the 21 RBPs. We observed a significant enrichment for miR-124, which presented the highest number of targets: six RBPs in total (*p*-value = 0.0099; hypergeometric test; Additional file 2: Figure S3).

We also evaluated whether the 21 RBPs presented mutations and/or copy-number alterations (CNA) in GBM samples from TCGA. We analyzed 273 GBM samples with exome sequencing and CNA data available in cBioPortal [45, 46]. Only 10 % of the samples displayed alterations in at least one of our selected RBPs. CNA, missense mutations, and/or truncating mutations were present in 17 out of 21 evaluated RBPs, not different from randomly selected RBPs sets (*p*-value > 0.1; simulation with 100,000 sets of 21 randomly selected RBPs; Additional file 2: Figure S4).

### RBPs impact cellular viability, proliferation, and apoptosis in GBM

The 21 selected RBPs were then evaluated in a functional screening. Transient knockdowns were performed with siRNAs (median knockdown efficiency ~90 %; Additional file 1: Table S6) in U251 and U343 GBM cells and their impact on viability (MTS assay), proliferation (IncuCyte), and apoptosis (Caspase-3/7 assay) were evaluated. Results of these three assays are summarized in Table 1 and represented in Additional file 2: Figures S5–S7. Out of the 21 investigated RBPs, 15 showed significant effect in at least one assay in one or both cell lines.

**Table 1:** Summary of functional assays results

#	Ensemble ID	Gene symbol	Viability (MTS)		Proliferation (IncuCyte)		Apoptosis (Caspase-3/7)	
			U251	U343	U251	U343	U251	U343
1	ENSG00000106305	*AIMP2*	✓	✓	-	-	-	-
2	ENSG00000183684	*ALYREF*	-	-	-	-	✓	-
3	ENSG00000179218	*CALR*	-	-	-	-	-	-
4	ENSG00000174371	*EXO1*	-	-	-	-	-	-
5	ENSG00000172183	*ISG20*	-	-	✓	-	-	-
6	ENSG00000162385	*MAGOH*	-	-	-	-	✓	-
7	ENSG00000111196	*MAGOHB*	-	-	-	-	✓	-
8	ENSG00000128626	*MRPS12*	-	-	-	-	-	-
9	ENSG00000090263	*MRPS33*	-	✓	-	✓	✓	-
10	ENSG00000132603	*NIP7*	✓	-	✓	✓	-	-
11	ENSG00000132661	*NXT1*	-	✓	✓	-	✓	-
12	ENSG00000111331	*OAS3*	-	-	-	-	-	-
13	ENSG00000171960	*PPIH*	-	-	-	-	-	-
14	ENSG00000153250	*RBMS1*	✓	✓	✓	✓	-	-
15	ENSG00000104889	*RNASEH2A*	✓	✓	✓	✓	✓	-
16	ENSG00000026297	*RNASET2*	-	-	-	-	-	-
17	ENSG00000175792	*RUVBL1*	✓	-	✓	-	✓	-
18	ENSG00000125835	*SNRPB*	✓	✓	✓	✓	✓	✓
19	ENSG00000143977	*SNRPG*	-	-	✓	-	✓	-
20	ENSG00000060138	*YBX3*	-	-	-	-	✓	-
21	ENSG00000088930	*XRN2*	-	✓	✓	-	-	-

✓ = significant difference compared to control (*p*-value < 0.05)

- = no significant difference compared to control (*p*-value ≥ 0.05)

### *SNRPB* as a potential new oncogenic candidate in GBM

**Fig. 3:**
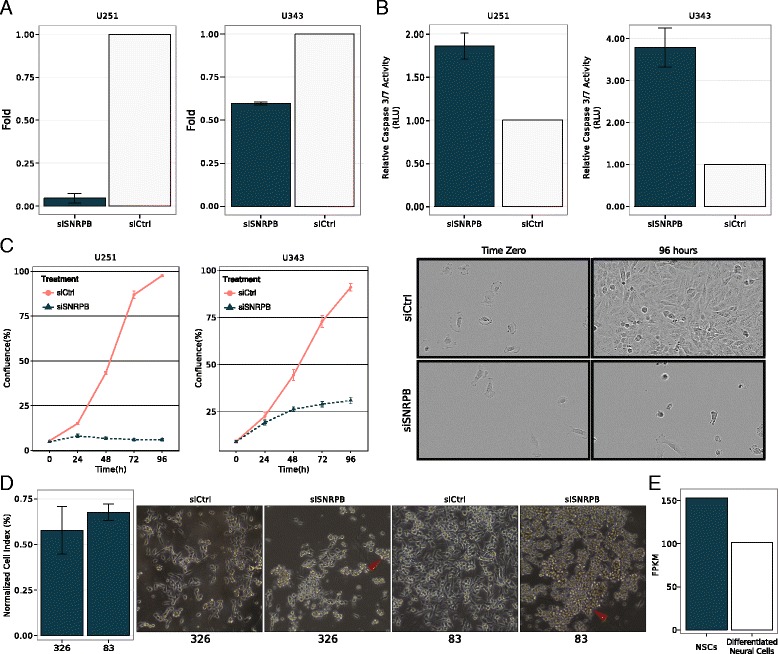
*SNRPB* impacts cancer-relevant processes. **a** Cellular viability was assayed by MTS and shows that *SNRPB* knockdown caused a significant reduction of viability in U251 (*p*-value = 0.002; Wilcoxon rank-sum test) and U343 cells (*p*-value < 0.001; Wilcoxon rank-sum test), compared to control transfected cell lines (*siCtrl*). **b** Caspase-3/7 assay shows an increase in apoptosis in siSNRPB compared to siCtrl in both cell lines (U251 *p*-value = 0.011; U343 *p*-value = 0.007; Wilcoxon rank-sum test). **c** Plots show confluence percentage monitored by IncuCyte over 96 hours, comparing siSNRPB with siCtrl. Proliferation reduction was observed in both cell lines upon *SNRPB* knockdown (U251 *p*-value < 0.001; U343 *p*-value < 0.001; ANOVA). *Right* panel shows U251 cellular profile at time zero and after 96 hours of transfection with siCtrl and siSNRPB. *SNRPB* knockdown resulted in a strong reduction in cell proliferation compared to control. **d** Downregulation of *SNRPB* by siRNA leads to inhibition of cell growth and cell detachment in two glioma stem cell lines. The percentage of the normalized cell index was calculated with respect to control. Results represent an average of two (line 326) or three (line 83) [39] individual experiments and each experiment was done in triplicate. *Red arrowheads* indicate the floated round up cell colonies on the plates where *SNRPB* siRNA was transfected. **e** *SNRPB* expression in mouse neural stem cells (*NSCs*) was compared to differentiated neural cells after 4 days by RNA-Seq. *SNRPB* expression was higher in undifferentiated cells (log_2_ fold change = 0.599347, FDR-adjusted *p*-value < 0.05)

Overall, *SNRPB*, which encodes core spliceosome components SmB/B’, exhibited the most consistent results in the functional screening: knockdown of this gene decreased viability (Fig. 3a), increased apoptosis (Fig. 3b), and decreased proliferation (Fig. 3c) in both U251 and U343 cell lines.

We conducted additional experiments to determine the impact of *SNRPB* on the growth of GSC cultures. Lines 326 and 83 were described in a previous study [39]. We knocked down the expression of *SNRPB* in these two GSC lines grown as conditionally reprogrammed cells (CRCs). CRCs have been shown to better recapitulate the characteristics of original tumor cells [47]. In both cell lines, *SNRPB* knockdown led to inhibition of cell growth and to cell detachment (Fig. 3d). Additionally, because GBMs are known to be highly undifferentiated tumors [48], we checked *SNRPB* expression in mouse neural stem cells versus differentiated neural cells and determined that *SNRPB* expression was higher in undifferentiated cells (Fig. 3e).

### *SNRPB* knockdown impacts the expression and processing of RNA splicing machinery components

To assess the contribution of *SNRPB* to GBM development, we performed its knockdown (Additional file 2: Figure S8) followed by RNA-Seq analysis in U251 cells. We then mapped changes in transcriptomic profiles and splicing events compared to control samples.

At the expression level, we found 7118 differentially expressed genes (log_2_ fold change > |1| and Benjamini-Hochberg corrected *p*-value < 0.05) upon *SNRPB* knockdown (3171 upregulated and 3947 downregulated genes; Additional file 1: Table S7). Among the upregulated genes, we observed strong enrichment for biological processes related to RNA processing and metabolism, splicing, and several cellular processes like differentiation, development, proliferation, migration, and signal transduction (Additional file 2: Figure S9A; Additional file 1: Table S8). Downregulated genes were enriched for processes related to DNA repair, DNA metabolism and replication (Additional file 2: Figure S9B; Additional file 1: Table S8).

At the splicing level, we found that 18,105 splicing events were altered upon *SNRPB* knockdown (difference in percentage spliced in (ΔPSI) > |0.1| and FDR-adjusted *p*-value < 0.05), affecting a total of 5692 genes. Events were classified in five categories: exon skipping (SE), mutually exclusive exons (MXE), alternative 5′ splice site (A5SS), alternative 3′ splice site (A3SS), or intron retention (RI). A summary showing results classified by event type is presented in Additional file 1: Table S9.

**Fig. 4:**
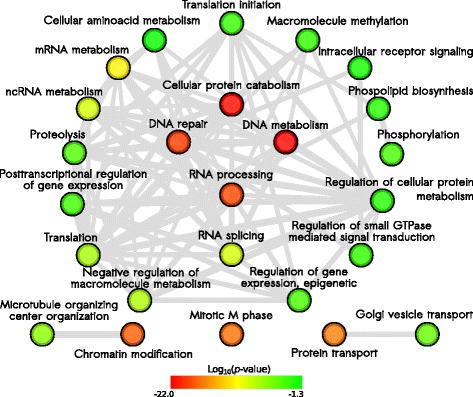
Gene Ontology (*GO*) annotation of genes presenting splicing alterations upon *SNRPB* knockdown. Network shows interaction between GO terms. *Colors* represent node significance. Top enriched terms were related to RNA processing, DNA metabolism and repair, chromatin modification, and cellular protein catabolism

**Fig. 5:**
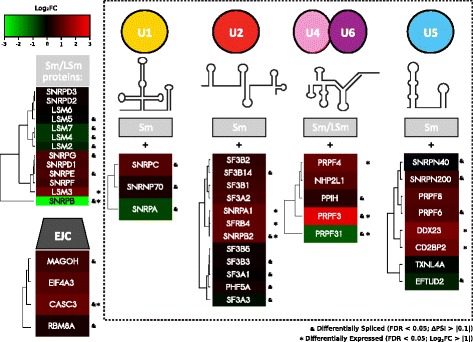
*SNRPB* knockdown impacts core spliceosome components. More than 20 % of the core spliceosome components were differentially expressed upon *SNRPB* knockdown; meanwhile, almost 60 % of them presented splicing alterations. *EJC* exon-junction complex, *ΔPSI* percentage spliced in. Adapted from [78, 114]

Similar to what was observed in the transcriptomic analysis, we determined that genes affected at the splicing level by *SNRPB* knockdown are preferentially associated with biological processes such as RNA processing and metabolism, splicing, DNA metabolism, and DNA repair (Fig. 4; Additional file 1: Table S10). Additional cancer relevant processes like chromatin remodeling were also identified (Fig. 4; Additional file 1: Table S10). In the particular case of RNA processing and splicing, we determined that core members of the small nuclear ribonucleic proteins (snRNPs), U1, U2, U4/U6, and U5, were greatly affected by *SNRPB* knockdown, especially at the splicing level: almost 60 % of them exhibited splicing alterations, which represents a strong enrichment when this gene set is compared to all multi-exon genes presenting at least one read on exon-exon junctions (*p*-value = 5.521199e-13; hypergeometric test; Fig. 5). These results suggest that SNRPB coordinates the splicing of spliceosome components.

### *SNRPB* knockdown impacts expression and processing of cancer genes and pathways already associated with gliomagenesis

**Fig. 6:**
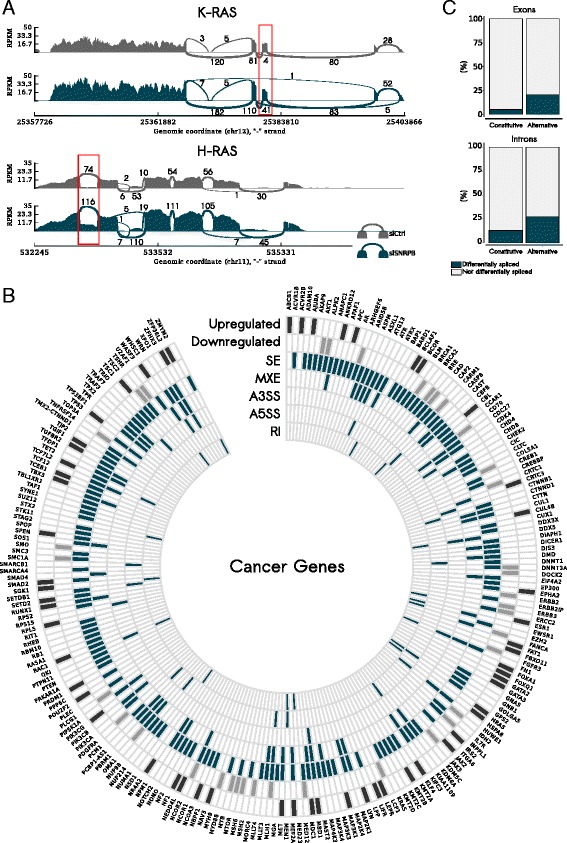
SNRPB impact on cancer genes and on alternative/constitutive exons/introns. **a** Sashimi plots highlighting two splicing events differentially regulated in siSNRPB compared with siCtrl: an exon skipping event in K-RAS (*top panel*) and an intron retention in H-RAS (*bottom panel*). **b** A total of 368 cancer genes were evaluated. Only genes presenting differential expression (upregulated or downregulated) or at least one splicing alteration [exon skipping (*SE*), mutually exclusive exons (*MXE*), alternative 5′ splice site (*A5SS*), alternative 3′ splice site (*A3SS*), or intron retention (*RI*)] are shown. **c** Alternative exons and introns are more affected by *SNRPB* knockdown than constitutive exons and introns

We also evaluated a set of 368 well-established cancer genes, manually curated from three different large-scale studies [49–51]. Out of 368 genes, 94 presented differential expression (57 upregulated and 37 downregulated). At the splicing level, ~50 % of the cancer genes presented at least one alteration. Enrichment for alterations at expression and splicing levels in this gene set were observed when compared to all expressed genes analyzed and all multi-exon genes presenting at least one read on exon-exon junctions, respectively (expression: *p*-value = 0.04123; splicing: *p*-value = 6.45815e-52; hypergeometric test; Fig. 6a, b).

We then specifically checked for alterations in genes involved in critical GBM pathways defined by TCGA: *RTK*, *PI3K*, *RAS*, *MAPK*, *AKT*, *RB*, and *p53* [6, 7]. All pathways were affected by *SNRPB* knockdown. At the expression level, 8 out of 33 evaluated genes were differentially expressed: four of them were upregulated (*HRAS*, *MET*, *NF1*, and *TP53*) and four downregulated upon knockdown (*AKT1*, *AKT2*, *FGFR3*, *PDGFRA*). No enrichment was observed when this category of genes was compared to all expressed genes exhibiting differential expression (*p*-value = 0.4250002; hypergeometric test). At the splicing level, 18 out of those 33 genes presented at least one differentially regulated splicing event (Additional file 2: Figure S10), showing strong enrichment for splicing alterations in this specific gene set when compared to all multi-exon genes having at least one read on exon-exon junctions (*p*-value = 5.409015e-07; hypergeometric test).

### Characteristics of exons/introns affected by *SNRPB* knockdown

SE and RI were two of the categories with the highest number of differentially regulated events and therefore were further investigated.

Knockdown of *SNRPB* reduced the inclusion levels of several exons (12,030 events with exons more included in control samples versus 462 events with exons more included in knockdown; Additional file 1: Table S8). Exons with higher exclusion levels upon knockdown were shorter than the ones with higher exclusion levels in control (median knockdown = 106 nucleotides, median control = 148 nucleotides; *p*-value < 2.2e-16; Wilcoxon-rank sum test; Additional file 2: Figure S11 left panel). With respect to GC content, exons whose inclusion in mature transcripts decreased upon *SNRPB* knockdown exhibited a lower percentage of GC when compared to the ones showing the opposite behavior (median knockdown = 47.25 %; median control = 50.57 %; *p*-value = 5.936e-07; Wilcoxon rank-sum test; Additional file 2: Figure S12 left panel). We also examined the strength of 3′ and 5′ splice sites (3′ss and 5′ss) associated with exons affected by *SNRPB* knockdown using the MaxEntScan approach [52]. Exons with higher exclusion levels upon knockdown were associated with stronger 3′ss and 5′ss compared to control (3′ss *p*-value < 2.2e-16; 5′ss *p*-value = 2.092e-14; Wilcoxon rank-sum test; Additional file 2: Figure S13 top panel).

Regarding introns, we observed that *SNRPB* knockdown favored the retention of introns in mature transcripts (835 intron retention events in knockdown compared to 116 in control samples; Additional file 1: Table S8). Introns showing increased retention in mature transcripts upon *SNRPB* knockdown were determined to be shorter than the ones preferentially retained in control samples (median knockdown = 483 nucleotides; median control = 1144 nucleotides; *p*-value < 2.2e-16; Wilcoxon rank-sum test; Additional file 2: Figure S11 right panel). Considering the GC content, introns more retained upon *SNRPB* knockdown presented a higher percentage of GC compared to the ones more retained in control (median knockdown = 59.07 %; median control = 43.96 %; *p*-value < 2.2e-16; Wilcoxon rank-sum test; Additional file 2: Figure S12 right panel). No significant difference was observed in the strength of 5′ss 3′ss associated with differentially regulated introns (3′ss *p*-value = 0.4464; 5′ss *p*-value = 0.9095; Wilcoxon rank-sum test; Additional file 2: Figure S13 bottom panel).

We also determined the effect of *SNRPB* knockdown on “constitutive” versus “alternative” exons and introns. Constitutive exons and introns were defined as those presenting with a PSI value = 1 and PSI = 0 in the control samples, respectively, whereas alternative exons and introns were defined as those with a PSI value < 1 (for exons) and PSI value > 0 (for introns) in control samples. In total, 5.6 % of the constitutive exons were affected by *SNRPB* knockdown, while 20.1 % of alternative exons showed changes (*p*-value < 2.2e-16; proportion test; Fig. 6c top panel). Regarding introns, 12.5 % of the constitutive ones were affected, while 26.4 % of the alternative introns presented alterations (*p*-value < 2.2e-16; proportion test; Fig. 6c bottom panel).

In summary, exons with higher exclusion levels upon *SNRPB* knockdown were shorter, and had lower GC content, and stronger 3′ss and 5′ss, whereas introns with higher retention levels upon knockdown were shorter, and exhibited higher GC content and no difference in 3′ss and 5′ss strength compared to the ones more retained in control samples.

## Discussion

Major changes in the expression of RBP coding genes are a noteworthy phenomena in multiple tumor tissues [16–18]. Here, we have corroborated this scenario in GBM. By comparing tumor samples (GBM samples from TCGA and GSC lines) to normal samples, we identified a set of 21 upregulated RBPs that also exhibited an impact on patient survival. We also found the expression levels of those RBPs was higher in GBMs than in lower grade gliomas, suggesting their potential impact on tumor progression and aggressiveness. To explore mechanisms that could contribute to the upregulation of those RBPs, we analyzed non-synonymous mutations, CNAs, and targeting by tumor suppressor miRNAs. We observed a modest contribution of mutations and CNAs. Because GBM does not exhibit a high mutational load compared to other tumor types [53], and has only 71 genes that are frequently mutated [7], the low number of samples harboring mutations in a small set of RBPs was expected. Regarding the tumor suppressor miRNAs, we found 18 of them targeting 15 out of 21 RBPs. Notably, miR-124 alone targets six RBPs. miR-124 is an important player in GBMs and impacts proliferation [54], tumor growth [55, 56], migration, and invasion [57].

The impact of RBP alterations in cancer is still poorly appreciated. One of the main reasons is that most available datasets include only mRNA expression levels, preventing any type of analysis to measure changes in splicing, mRNA decay, and translation. However, this scenario is improving, especially with the advent of functional genomics methods, like ribosomal profiling and more sensitive proteomics platforms. In addition, the transcriptomics field is moving away from microarray towards RNA-Seq, which provides an opportunity to investigate global changes in splicing [58]. Recent investigations of alternative splicing across multiple cancer types have revealed splicing as an important source of transcriptional diversity in many cancers and allowed the identification of a common set of cancer-specific splicing events, which can potentially be used as novel biomarkers with application in molecular diagnosis and prognosis [59–61].

We identified an interesting subset of aberrantly expressed RBPs implicated in splicing, pointing to an additional layer of alterations that could contribute to GBM development. Involvement of splicing proteins in cancer and other disorders is capturing the interest of the scientific community. One of the most studied cases is *SF3B1*, which is mutated in ~20 % of patients with myelodysplastic syndromes (MDS). Other splicing regulators, including *PRPF40B*, *SRSF2*, *SF3A1*, *U2AF1*, and *ZRSR2*, also display a high mutation frequency in MDS [62]. Studies of hematological malignancies showed similar results. For instance, *SF3B1*, *SRSF1*, *U2AF65*, and *CELF4* are often mutated in chronic lymphocytic leukemia [63]. Subsequent reports revealed that alterations in splicing factors occur in solid tumors, including neuroblastomas, pancreatic ductal adenocarcinoma, lung cancer, melanoma, colon cancer, and estrogen receptor-positive breast tumors [64, 65]. In GBM, the splicing factors PTB, hnRNP H and A2/B1, and WTAP have been shown to regulate several biological processes relevant to cancer development [29–34, 66]. Moreover, numerous examples of cancer-relevant genes affected at the splicing level (e.g. *ANXA7*, *GLI1*, *MAX*, *KLF6*) have been reported in GBM [30, 31, 34, 67–70]. Besides contributing to tumorigenesis via splicing regulation, splicing factors can have additional routes of action. For instance, genomic instability, a common feature in cancer, can be induced by RNA processing defects [71].

*SNRPB*, which encodes core members of the spliceosome machinery, SmB/B’, was the main focus of our study. Its knockdown decreased viability, increased apoptosis, and decreased proliferation in both U251 and U343 cell lines. One would expect that alterations in core splicing proteins, such as the ones encoded by *SNRPB*, could cause major disruptions in RNA processing, affecting the entire transcriptome in a global and homogeneous manner. However, a different scenario has been observed, with splicing regulators impacting distinct sets of events when their expression levels are altered. For instance, in a recent study, 270 core splicing proteins and other RNA-related factors were systematically knocked down and the impact on splicing of 38 genes associated with proliferation and apoptosis was investigated [72]. It was observed that each splicing factor regulated a specific set of events, and factors involved in the same splicing step tended to affect the same group of events. Results were corroborated by RNA-Seq studies in which specific changes in splicing, mainly in alternative exons, were observed upon knockdown of core splicing proteins, including SNRPB [73–75].

In addition to its essential role in splicing, mutations in *SNRPB* are known to cause cerebro-costo-mandibular syndrome [76–78]. Furthermore, a screening for genes required for cell division revealed *SNRPB* along with other splicing factors [79]. However, *SNRPB* is relatively poorly characterized in terms of contributions to tumorigenesis. Its expression is altered in non-small cell lung cancer along with other genes involved in RNA metabolism and is correlated with a poor prognosis [80]. In a mouse allograft model of prostate cancer (NE-10), *SNRPB* was identified as a candidate metastasis suppressor gene [81]. Quantitative expression analysis confirmed decreased expression of SNRPB in the metastasizing compared to non-metastasizing tumors [81]. These data along with ours suggest that SNRPB can have different roles in tumorigenesis depending on context.

Alternative splicing events can result in transcript isoforms with reading frame disruption, lower stability, and improper localization in comparison to constitutive isoforms. Our RNA-Seq analysis determined some trends in terms of splicing events upon *SNRPB* knockdown. Alternative exons and introns were more affected than the constitutive ones, suggesting SNRPB functions to help the recognition of exons and introns containing weaker regulatory elements, such as alternative exons.

Gene ontology enrichment analysis of gene expression and splicing data revealed that *SNRPB* influences a large number of biological processes with relevance to cancer, such as RNA processing and DNA repair. Alterations in a large number of RNA processing/splicing genes places *SNRPB* as a central regulator and suggests that several of the splicing alterations we observed upon *SNRPB* knockdown might be in fact a secondary effect. Radio-resistance, which is largely influenced by genes in the DNA repair pathway, is a major problem in cancer treatment and it is particularly relevant to GBM. Splicing alterations have been described for a large number of DNA repair genes, including several that we determined to be influenced by SNRPB levels (*BRAC1*, *BARD1*, *MSH2*, *RAD50*, *CHEK1*) [82–86]. Additionally, we observed that knockdown of *SNRPB* altered multiple genes associated with critical genes/pathways relevant to GBM development (*RTK*, *PI3K*, *MAPK*, *RAS*, *AKT*, *RB*, and *p53*) and other cancer genes.

## Conclusion

Despite the need for a more detailed analysis to determine how alterations identified here affect protein function in specific ways to contribute to tumor initiation and growth, we conclude that our data suggest diverse routes by which SNRPB influences GBM development.

## Methods

### Gene expression analysis of GBM RNA-Seq data from TCGA

RNA-Seq raw reads from 170 samples of GBM from TCGA [87] were downloaded from Cancer Genomics Hub (CGHub [88]; Additional file 1: Table S1). Normal brain samples were downloaded from the SRA [89] database [SRA: SRP028705 and ERP003613], Human Body Map [SRA: ERR030882], and TCGA (Additional file 1: Table S1). Reads were mapped against the human genome (version hg19/GRCh37 – UCSC Genome Browser [90]) using GSNAP [91] (version 2014-05-15). Mapped reads with quality (Q) ≥ 20 (Phred scale) were selected using SAMtools [92]. Read counts per gene were defined using HTSeq [93] and GENCODE (version 19 [15]) as the reference transcriptome. Differential expression analysis was performed using DESeq2 [94] comparing tumor samples to normal samples. All genes differentially expressed between tumor and normal samples (Benjamini-Hochberg corrected *p*-value < 0.05 and log_2_ fold change ≥ |1|) were selected. The catalog containing 1542 human RBPs from Gerstberger et al. [14] was used as a reference to identify all differentially expressed RBPs.

### Gene expression analysis of GSCs microarray data

Microarray data (Affymetrix platform: Human U219) of 10 glioma stem cell lines and one normal neural progenitor cell line, in triplicate, were obtained from Mao et al. [39]. Data were normalized using Robust Multichip Average (RMA; Affy package [95]). Differentially expressed RBPs between normal and GSC samples (Benjamini-Hochberg corrected *p*-value < 0.05) were identified using the LIMMA package [96].

### Survival analysis

The REMBRANDT dataset (REpository for Molecular BRAin Neoplasia DaTa [42]) was used to evaluate whether increased expression of the selected RBPs was associated with a poorer prognosis in brain neoplasia. Samples with increased expression of selected RBPs (log_2_ fold change ≥ 1) were compared to all other samples. Kaplan-Meier survival curves were built and then compared using a log-rank test. Differences resulting in a *p*-value < 0.05 were considered significant.

### Comparison of normal brain samples from GTEx with GBM samples from TCGA

Read counts per gene of 222 samples from normal brain (cortex and frontal cortex) were downloaded from the GTEx portal [43]. Those samples were compared to 170 GBM samples from TCGA. Read counts per gene of GBM samples were generated as described previously. Differential expression analysis was performed using DESeq2 [94], comparing tumor to normal samples, and the expression levels of 21 RBPs were analyzed. RBPs presenting Benjamini-Hochberg corrected *p*-values < 0.05 were considered to be differentially expressed.

### RBPs expression in lower and higher grade gliomas

Level 3 normalized expression data from 167 grade IV gliomas (GBMs) and 218 lower grade gliomas (LGG: 31 grade II astrocytomas, 73 grade II oligodendrogliomas, 68 grade III astrocytomas, and 46 grade III oligodendrogliomas) were downloaded from TCGA [87]. Expression levels of 21 RBPs in LGG were compared with GBM samples using Wilcoxon rank-sum test.

### Mutation and CNA analysis

All 273 GBM samples with exome sequencing and CNA data available in cBioPortal [45, 46] were evaluated (dataset: Glioblastoma Multiforme – TCGA, Provisional). The gene set containing 21 selected RBPs was analyzed and all samples containing at least one alteration in one or more of these RBPs were identified and presented. A simulation with 100,000 random sets of 21 out of 1542 RBPs was performed to determine if our selected set presented enrichment for CNA and mutations. Mutation and CNA data for all RBPs were retrieved from cBioPortal using the CDGS-R package [97].

### Tumor suppressor miRNAs targeting RBPs

A list containing frequently downregulated miRNAs in GBM (tumor suppressor miRNAs) was downloaded from Hermansen and Kristensen [44]. We then used the miRTarBase database [98] to select all genes targeted by those tumor suppressor miRNAs. Next, we identified which of those miRNAs target at least one of the 21 selected RBPs. Enrichment was calculated using a hypergeometric test.

### Functional annotation

Functional annotation analyses (Gene Ontology and KEGG pathways) were performed using DAVID [99], using *Homo sapiens* genes as background. Terms with Benjamini-Hochberg corrected *p*-values < 0.05 were determined as enriched. Redundant GO terms were summarized using REViGO [100]. Networks of GO terms were built using Cytoscape [101].

### Cell growth and transfection

U251 and U343 GBM cells (from American Type Culture Collection, Manassas, VA, USA) were grown in Dulbecco’s Modified Eagle medium with 10 % fetal bovine serum. Cells were synchronized through serum starvation for 48 hours. siRNAs (ON-TARGETplus SMARTpool; Dharmacon) for 21 RBPs and one siRNA control were transfected using Lipofectamine RNAiMax reagent (Invitrogen) according to the manufacturer’s instructions. All following experiments were performed in triplicate.

We established serum-free 3D cultures from two individual GSC lines (326 and 83) previously obtained by Dr Ichiro Nakano [39] during his time at The Ohio State University. Information regarding the Human Protocol and patient consent are described in the original publication [39]. Cells were trypsinized using TrypLE (Invitrogen) and plated in a collagen-coated T-25 flask with 10,000 irradiated (3000 rad) human mesenchymal stem cells (Lonza) in a conditionally reprogrammed FY medium [3:1 (v/v) F-12 Nutrient Mixture (Ham)/Dulbecco’s Modified Eagle medium (Invitrogen), 5 % fetal bovine serum, 0.4 μg/mL hydrocortisone (Sigma-Aldrich), 5 μg/mL insulin (Sigma-Aldrich), 8.4 ng/mL cholera toxin (Sigma-Aldrich), and 10 ng/mL epidermal growth factor (Invitrogen)] with the addition of 5 μmol/L Y-27632 (Enzo Life Sciences) [47]. Cells were grown in a humidified incubator at 37 °C with 5 % carbon dioxide for several passages to ensure the stability of cultures. For knockdown experiments, 200,000 GSC cells were plated in a collagen-coated six-well plate along with 2000 irradiated human mesenchymal stem cells in conditionally reprogrammed cell media containing FY medium. The next day, 25 nM of either scrambled or *SNRPB* siRNA suspended in RNAiMAX was added to the wells. Subsequently, each well was washed twice with phosphate-buffered saline and 500 μL of OPTIMEM was added. After 5–6 hours, 2 mL of conditionally reprogrammed media was incorporated into each well. After 72 hours, the floating cell fraction was collected and mixed with trypsinized attached cells from each well. Cell counting was performed using a Countess automated cell counter (Life Technologies) according to the manufacturer’s protocol. Transfections were performed in triplicate and each experiment was done at least two times. Total RNA was isolated by pooling three wells from each experiment and using an RNeasy kit (Qiagen) according to the manufacturer’s instructions. The percentage normalized cell index for SNRPB-specific siRNA was calculated by normalizing the cell index with control siRNA. The standard deviation was calculated for each experiment and then averaged to obtain cumulative standard deviation.

### Cell viability assay

After transfection, U251 and U343 cells were grown in 96-well cell culture plates. Cell viability was assessed by CellTiter 96 AQueous One Solution (Promega) reagent after 72 hours of incubation. Absorbance at 490 nm was quantified using the SpectraMax M5 microplate reader (Molecular Devices). Data were analyzed using Student’s *t*-test and presented as the relative mean ± standard error.

### Proliferation assay

After transfection, U251 and U343 cells were grown in 96-well cell culture plates. The confluence percentage was monitored for 96 hours using a high-definition automated imaging system (IncuCyte; Essen BioScience). Data were evaluated using ANOVA and presented as mean ± standard error.

### Caspase-3/7 apoptosis assay

U251 and U343 cells were grown in 96-well plates after transfection. After 72 hours of incubation, apoptosis levels were assessed using the Caspase-Glo 3/7 assay kit (Promega), according to the manufacturer’s protocol. Luminescence was measured using the SpectraMax M5 microplate reader (Molecular Devices). Data were analyzed using Student’s *t*-test and presented as mean of relative light units ± standard error.

### Gene expression analysis of RNA-Seq data from neural stem cells

RNA-Seq data from mouse neural stem cells and differentiated cells after 4 days [GEO: GSE67135] was used to analyze expression levels of *SNRPB* in both conditions. The HomoloGene database [102] was used to identify *SNRPB* orthologs between human and mouse. *SNRPB* gene expression in undifferentiated cells was compared to its expression in differentiated neural cells.

### Knockdown quantification by real-time PCR

Total RNA was extracted using TRIzol reagent (Invitrogen) according to manufacturer’s instructions. Reverse transcription of messenger RNAs was performed using a high-capacity cDNA reverse transcription kit (Applied Biosystems) with random priming. For mRNA analysis, quantitative PCR was performed using the primers listed in Additional file 1: Table S6 and Power SYBR Green PCR Master Mix (Applied Biosystems). Real-time PCRs were performed on the ViiA™ 7 Real-Time PCR System (Applied Biosystems). Data were acquired using the ViiA 7 RUO software (Applied Biosystems) and analyzed using the 2^−ΔΔCT^ method with GAPDH as an endogenous control.

### Knockdown quantification by western blot

Cells were resuspended and sonicated in Laemmli sample buffer, separated on a 13.5 % sodium dodecyl sulfate polyacrylamide gel electrophoresis (SDS-PAGE) gel, and transferred to polyvinylidene fluoride (PVDF) membranes. After transfer, membranes were blocked in Tris-buffered saline with Tween 20 and 5 % milk. Membranes were probed with rabbit anti-α-SNRPB (GeneTex; dilution 1:500) and mouse anti-α-tubulin antibody (Sigma; dilution, 1:2000). Horseradish peroxidase (HRP)-conjugated goat anti-rabbit antibody (Santa Cruz Biotechnology) or HRP-conjugated goat anti-mouse antibody (Zymed Laboratories, Carlsbad, CA, USA) were used as secondary antibodies. Electrochemiluminescence was used to detect the selected proteins using Immobilon Western chemiluminescent substrate (Millipore, Billerica, MA, USA).

### RNA preparation and sequencing

U251 cells were transiently transfected with control or SNRPB siRNAs using Lipofectamine RNAiMAX (Invitrogen). The experiment was performed in triplicate. Knockdown levels of SNRPB were ~90 %, as measured by quantitative reverse transcription-PCR (qRT-PCR). Total RNA was extracted using the TRIzol reagent (Life Technologies) and further purified with RNeasy (Qiagen), according to manufacturer’s instructions. Samples were prepared for RNA-Seq according to Illumina instructions and sequenced in a HiSeq-2000 machine by UTHSCSA Genomic Facility.

### Alternative splicing analysis

To identify splicing alterations produced by *SNRPB* knockdown, raw RNA-Seq reads of control and knockdown experiments were mapped against the human reference genome (hg19/GRCh37) and a reference transcriptome (GENCODE version 19 [15]) using GSNAP [91] (version 2014-05-15). Next, only reliable alignments (Q ≥ 20; Phred-scale) were selected using SAMtools [92]. Multivariate Analysis of Transcript Splicing (MATS [103, 104]) was used to search for splicing differences between *SNRPB*-knockdown and control samples. Only those isoforms differentially represented between conditions (FDR-adjusted *p*-value < 0.05 and ΔPSI > |0.1|) were selected. Splicing variants were classified as SE, MXE, RI, alternative donor site (A5SS), or alternative acceptor site (A3SS). 3′ss and 5′ss strengths of the differentially spliced exons and introns were calculated using the MaxEntScan approach [52].

### Statistical analysis and figures

All statistical analyses were executed using R [105]. Figures were built using R [105], Cytoscape [101], Circos Plot [106], Sashimi plot [107], and Inkscape [108].

## Additional files

Additional file 1: Contains supplementary **Tables S1–S10.** **Table S1.** GBM RNA-Seq samples from TCGA and Normal samples from SRA and TCGA. **Table S2.** Differentially expressed RBPs in GBM data from TCGA. **Table S3.** Differentially expressed RBPs in GSCs data. **Table S4.** RBPs expression in GBM samples from TCGA vs. normal brain samples from GTEx. **Table S5.** Wilcoxon rank-sum test results: GBM vs. lower grade gliomas. **Table S6.** Knockdown efficiency measured by qRT-PCR. **Table S7.** Differentially expressed genes upon *SNRPB* knockdown. **Table S8.** Functional annotation of differentially expressed genes upon *SNRPB* knockdown. **Table S9.** Summary of differentially regulated splicing events upon *SNRPB* knockdown. **Table S10.** Functional annotation of differentially spliced genes upon *SNRPB* knockdown. (XLS 822 kb) Additional file 2: Contains supplementary **Figures S1–S13.** (PDF 4857 kb)
