# Supplementary material for: Functional genomics analyses of RNA-binding proteins reveal the splicing regulator SNRPB as an oncogenic candidate in glioblastoma
Source: Genome Biol. 2016 Jun 10;17:125. doi: 10.1186/s13059-016-0990-4 (PMC4901439; doi:10.1186/s13059-016-0990-4)
Supplement: Additional file 2: — Contains supplementary Figures S1–S13. (PDF 4857 kb) [file 13059_2016_990_MOESM2_ESM.pdf]

# Additional File 2

Supplementary Figures

# Functional genomics analyses of RNA-binding proteins reveal the splicing regulator SNRPB as an oncogenic candidate in glioblastoma

Bruna R. Correa; Patricia Rosa de Araujo; Mei Qiao; Suzanne C. Burns; Chen Chen; Richard Schlegel; Seema Agarwal; Pedro A. F. Galante; Luiz O. F. Penalva

**Table of Contents**

**Figure S1. Differentially expressed RBPs by GBM subtype.** **Pg. 2**
**Figure S2. Kaplan-Meier survival plots.** **Pg. 3**
**Figure S3. RBPs regulated by tumor suppressor miRNAs.** **Pg. 6**
**Figure S4. Copy-number alteration and mutation analysis.** **Pg. 7**
**Figure S5. Viability assay by MTS.** **Pg. 8**
**Figure S6. Proliferation assay – U251 cell line.** **Pg. 9**
**Figure S7. Proliferation assay – U343 cell line.** **Pg. 10**
**Figure S8. Western blot of SNRPB knockdown.** **Pg. 11**
**Figure S9. Gene ontology annotation of differentially expressed genes.** **Pg. 12**
**Figure S10. SNRPB knockdown impacts genes/pathways associated with gliomagenesis.** **Pg. 13**
**Figure S11. Length of differentially spliced exons and introns.** **Pg. 14**
**Figure S12. GC content of differentially spliced exons and introns.** **Pg. 15**
**Figure S13. 3'ss and 5'ss strength of differentially spliced exons/introns.** **Pg. 16**

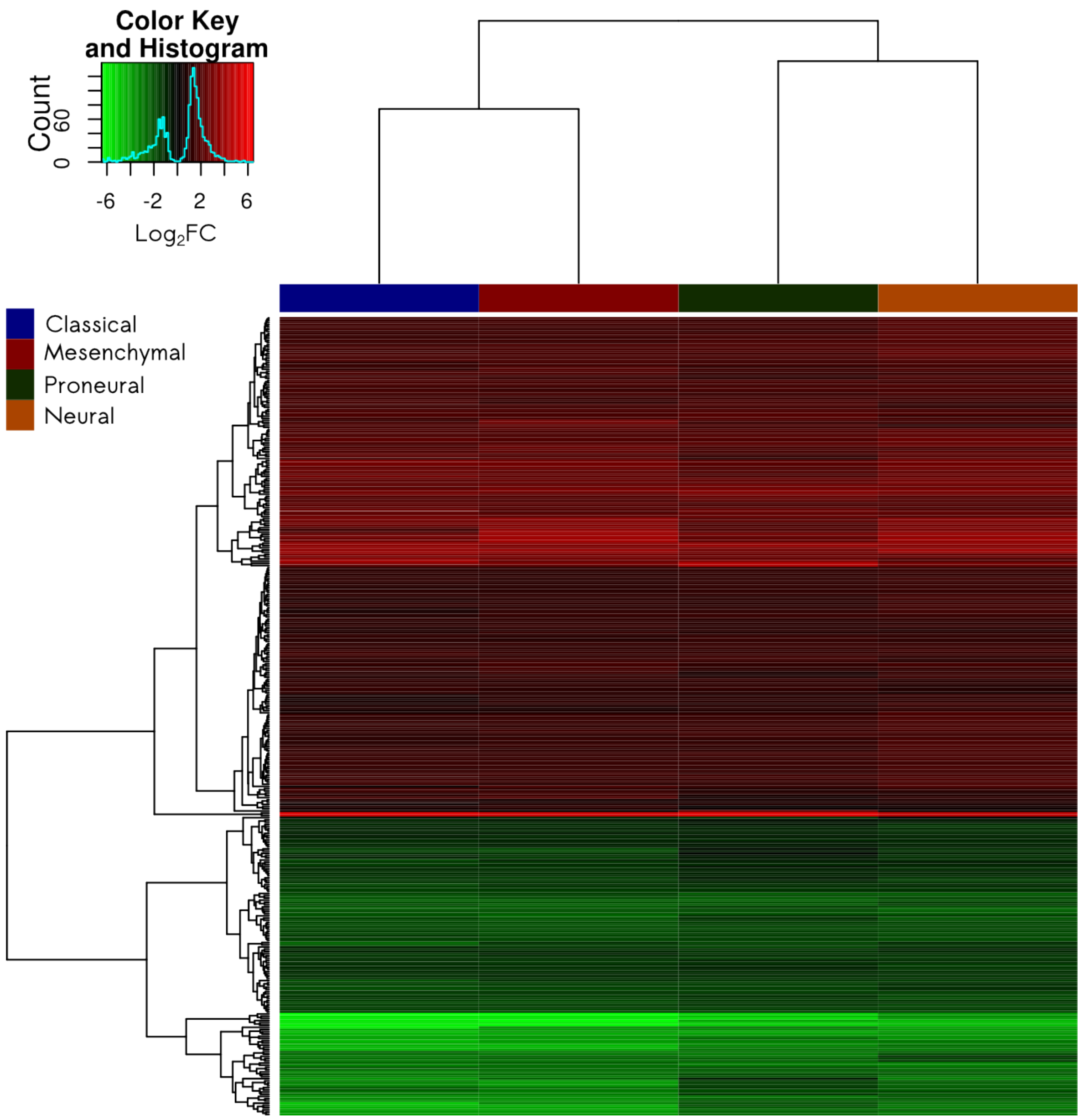


**Figure S1. Differentially expressed RBPs by GBM subtype**. 358 differentially expressed RBPs grouped according GBM subtypes defined by TCGA.

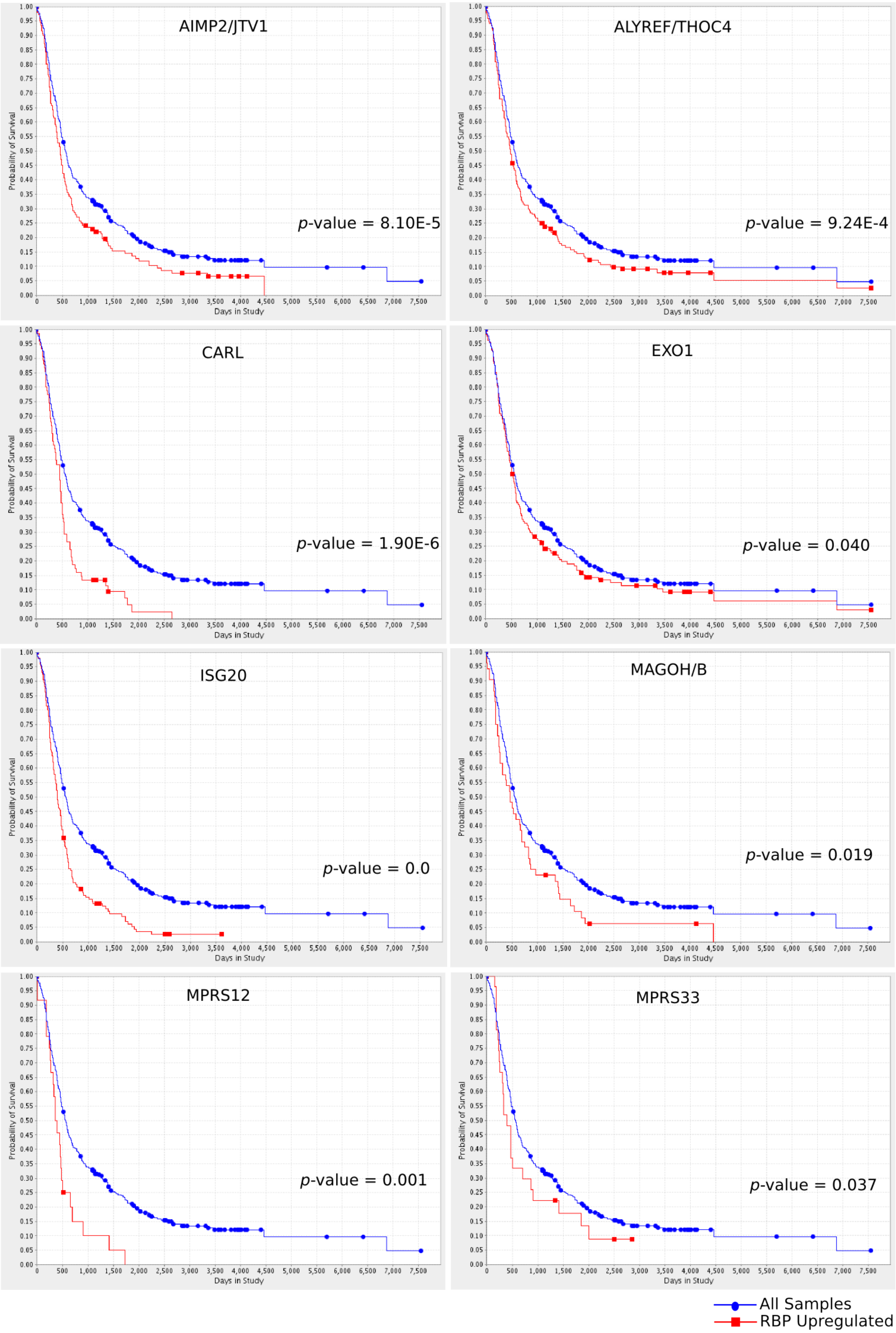


**Figure S2. Kaplan-Meier survival plots**. RBPs associated with survival reduction when upregulated are shown.

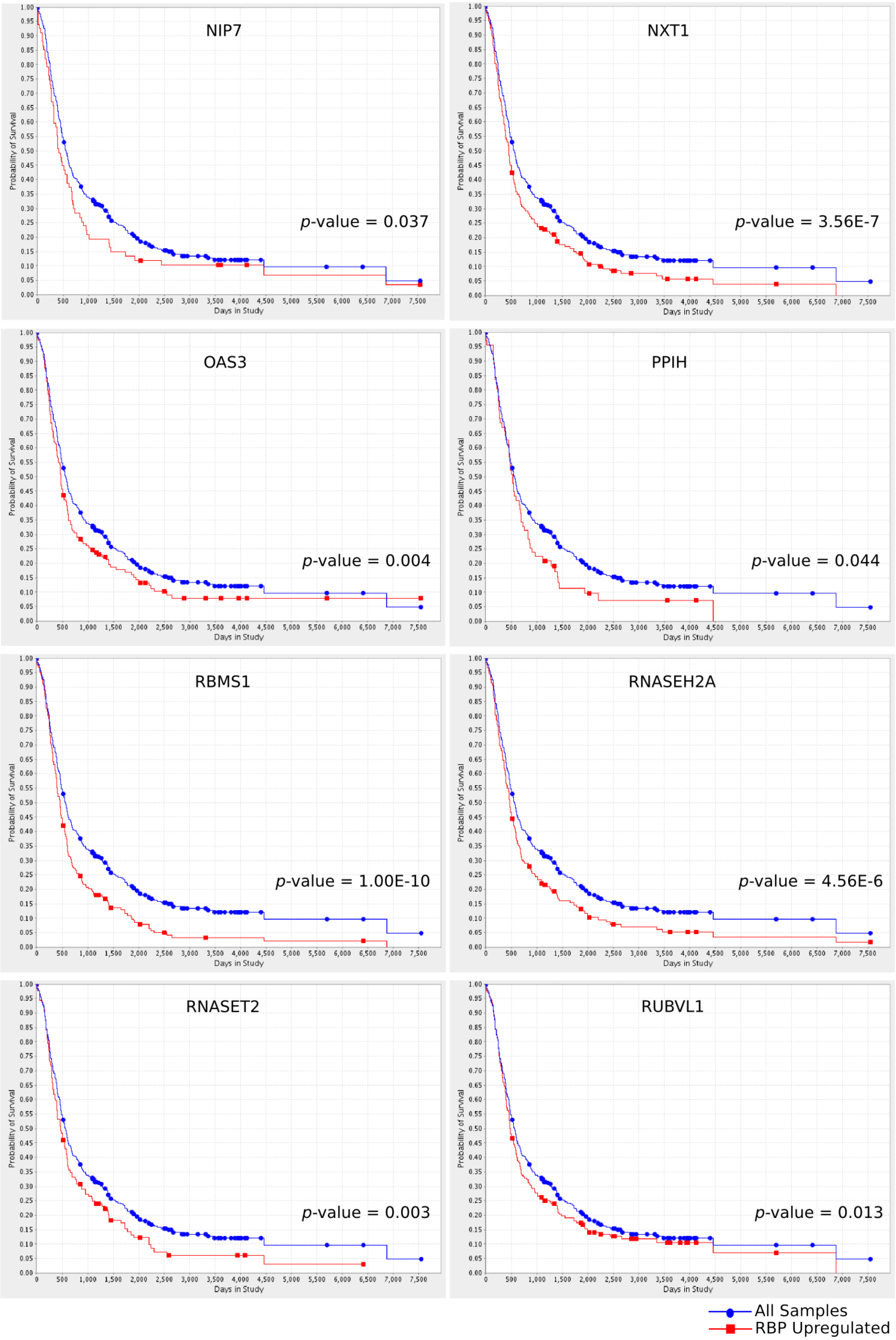


(cont.)**Figure S2. Kaplan-Meier survival plots.**

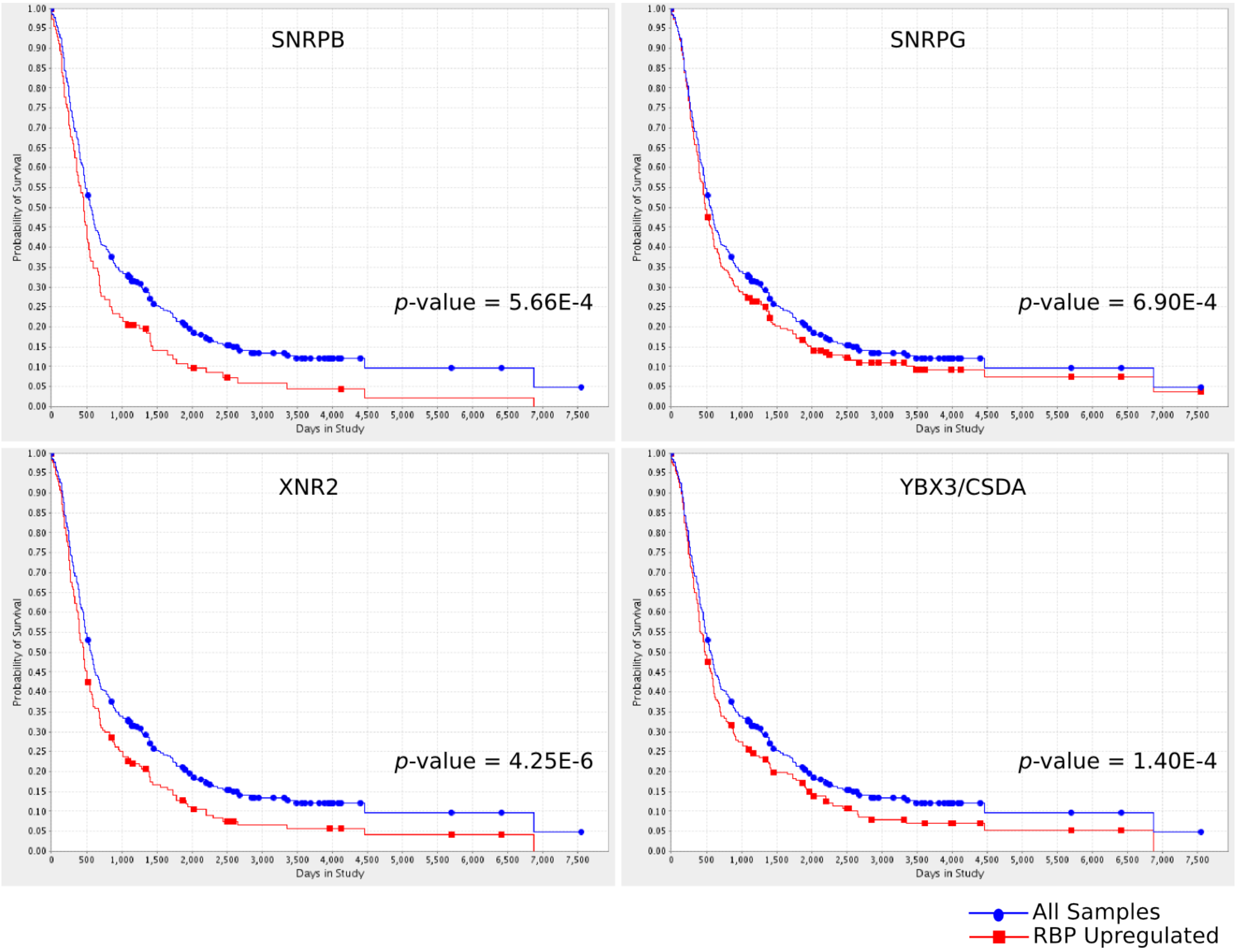


(cont.)**Figure S2. Kaplan-Meier survival plots**.

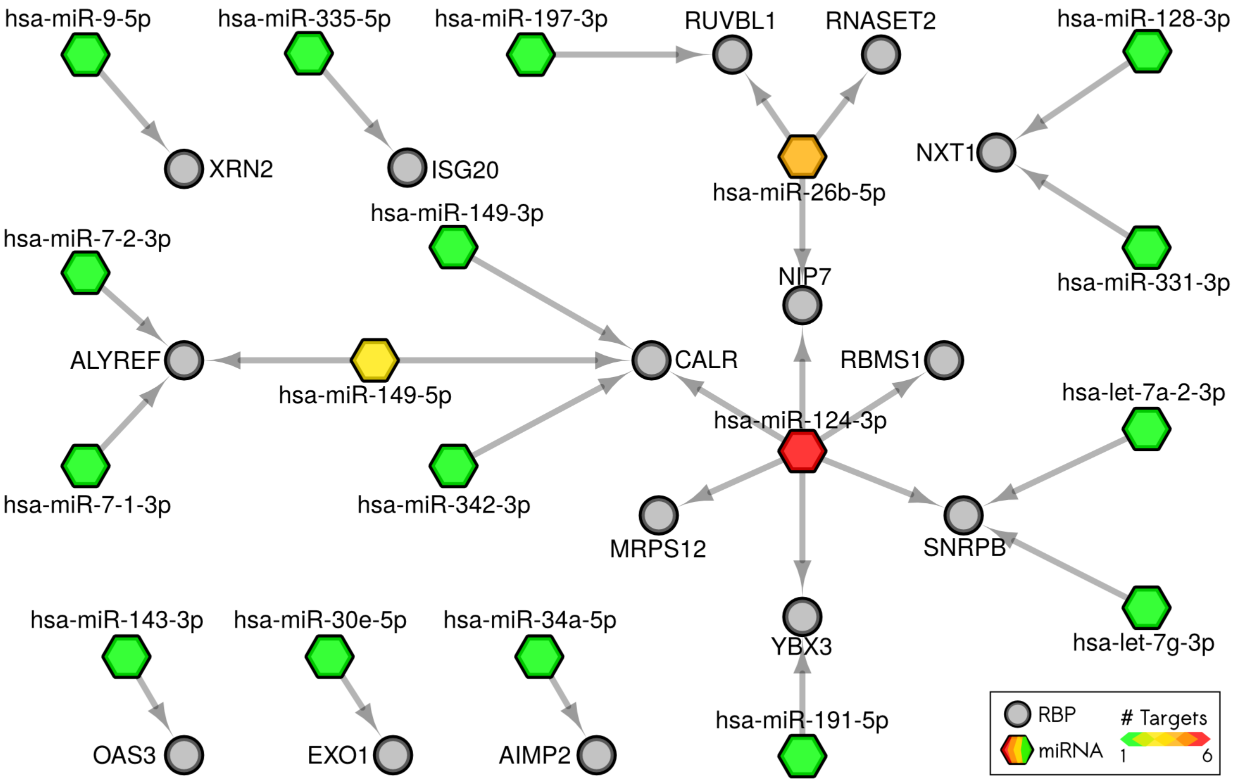


**Figure S3. RBPs regulated by tumor suppressor miRNAs.** 15 out of 21 selected overexpressed RBPs are targeted by 18 miRNAs frequently downregulated in GBMs. miR-124-3p alone targets 6 RBPs.

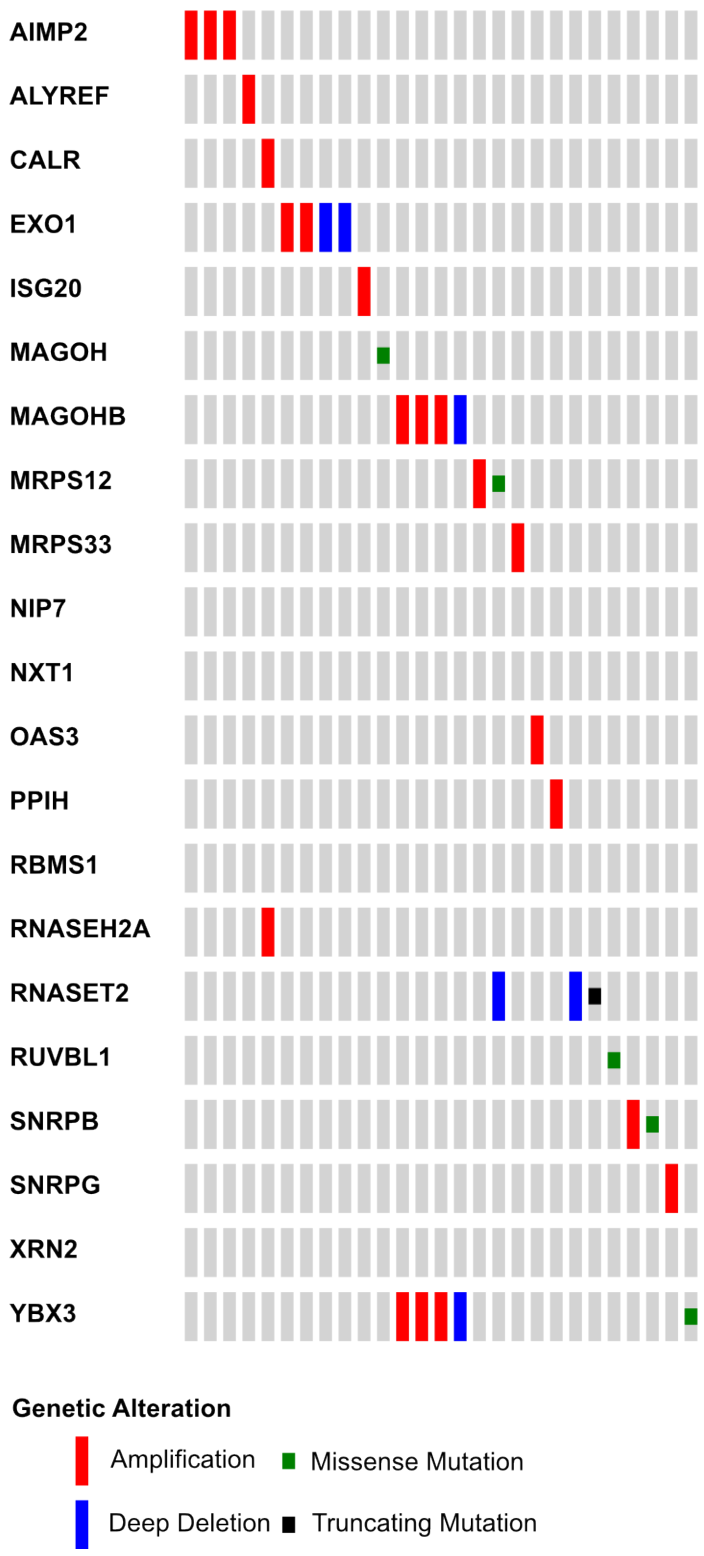


**Figure S4. Copy-number alteration and mutation analysis.** 273 GBM samples with exome sequencing and CNA data available in cBioPortal were analyzed, and alterations in our 21 selected RBPs were identified. Only samples presenting at least one alteration are shown.

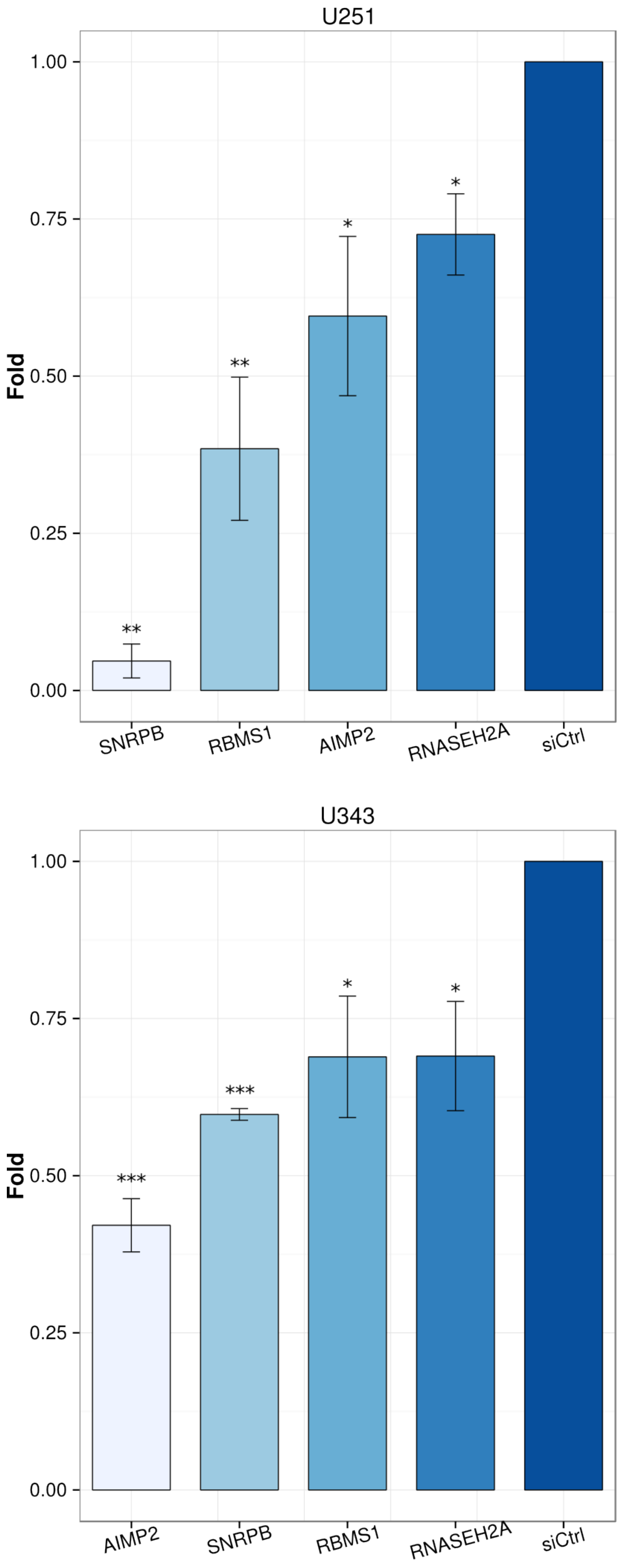


**Figure S5. Viability assay by MTS**. Only RBPs showing significant viability reduction in U251 and U343 cell lines (*p*-value < 0.05) upon knockdown are shown (* *p*-value < 0.05; ** *p*-value < 0.01; *** *p*-value < 0.001).

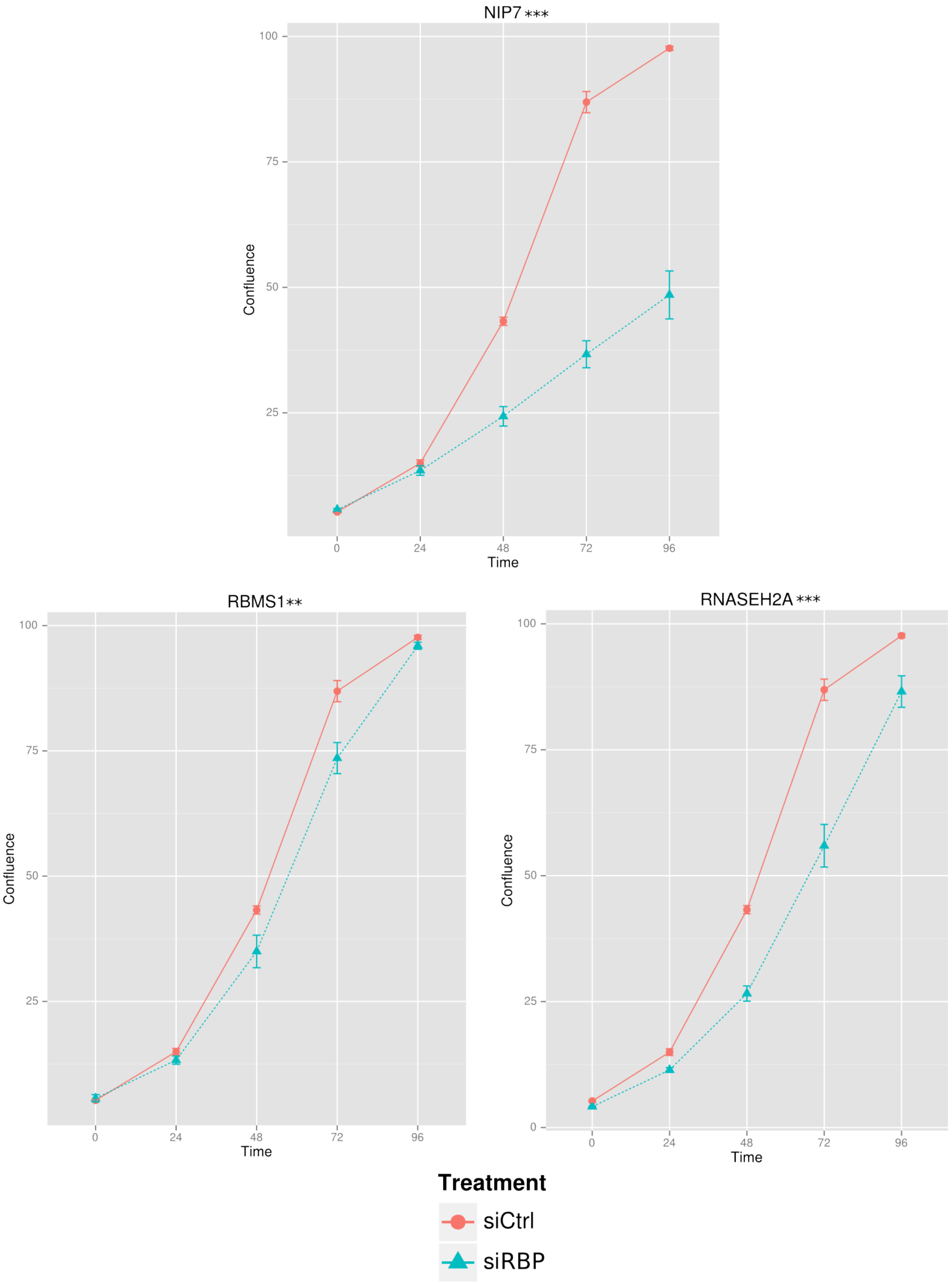


**Figure S6. Proliferation Assay – <u>U251</u> cell line**. Only RBPs showing significant reduction in proliferation capacity in both cell lines ($p$-value < 0.05) upon knockdown are shown (* $p$-value < 0.05; ** $p$-value < 0.01; *** $p$-value < 0.001).

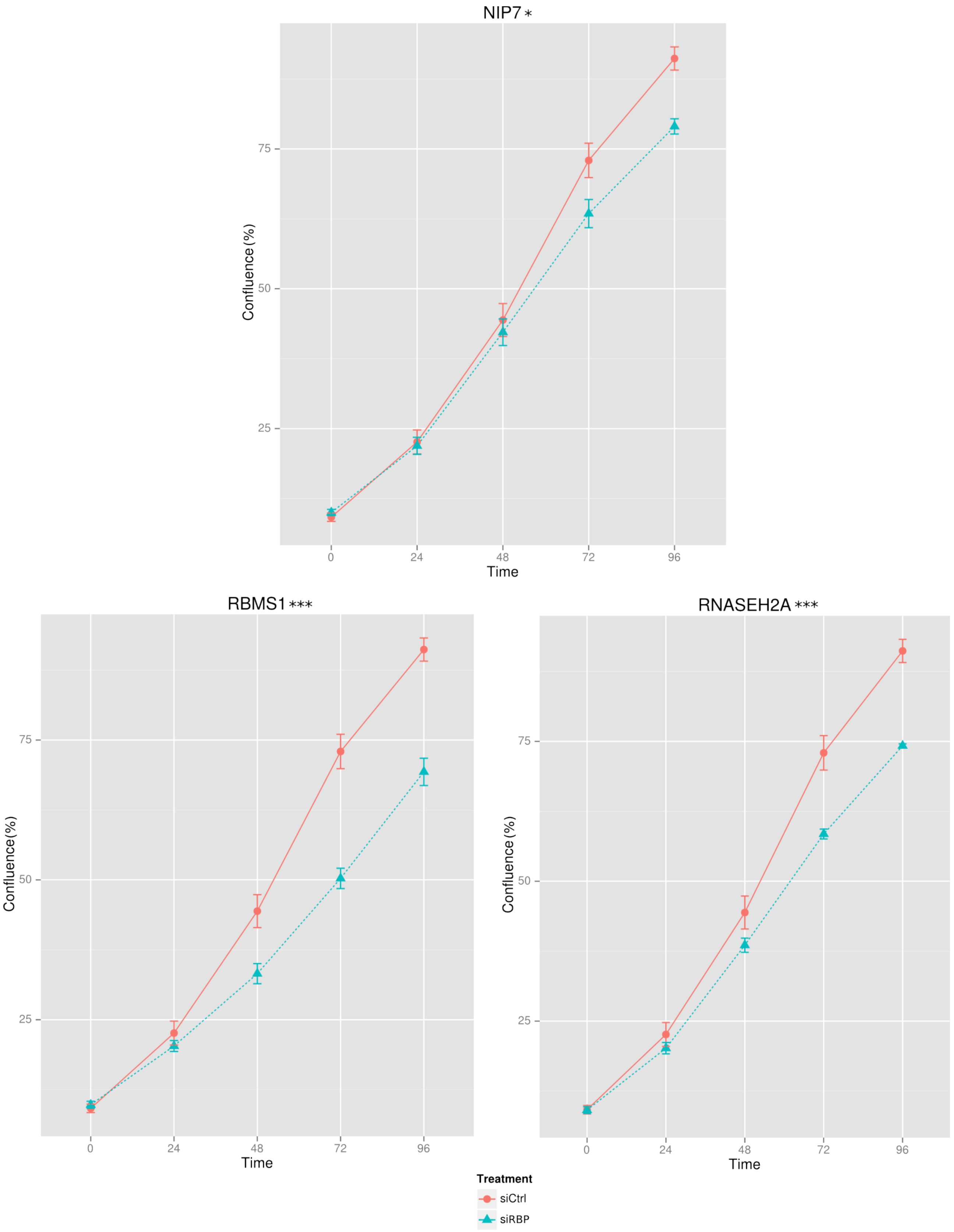


**Figure S7. Proliferation Assay – <u>U343</u> cell line**. Only RBPs showing significant reduction in proliferation capacity in both cell lines ($p$-value < 0.05) upon knockdown are shown (* $p$-value < 0.05; ** $p$-value < 0.01; *** $p$-value < 0.001).

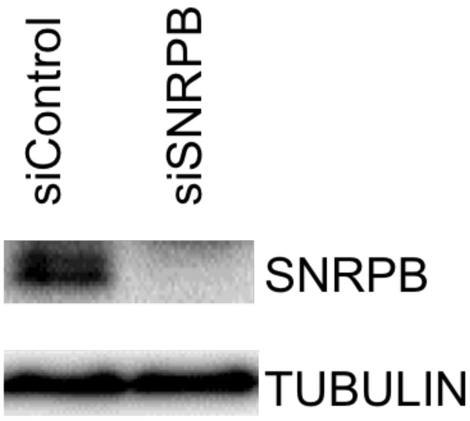


**Figure S8. Western blot of SNRPB knockdown.** Western analyses show the impact of knockdown on SNRPB protein levels.

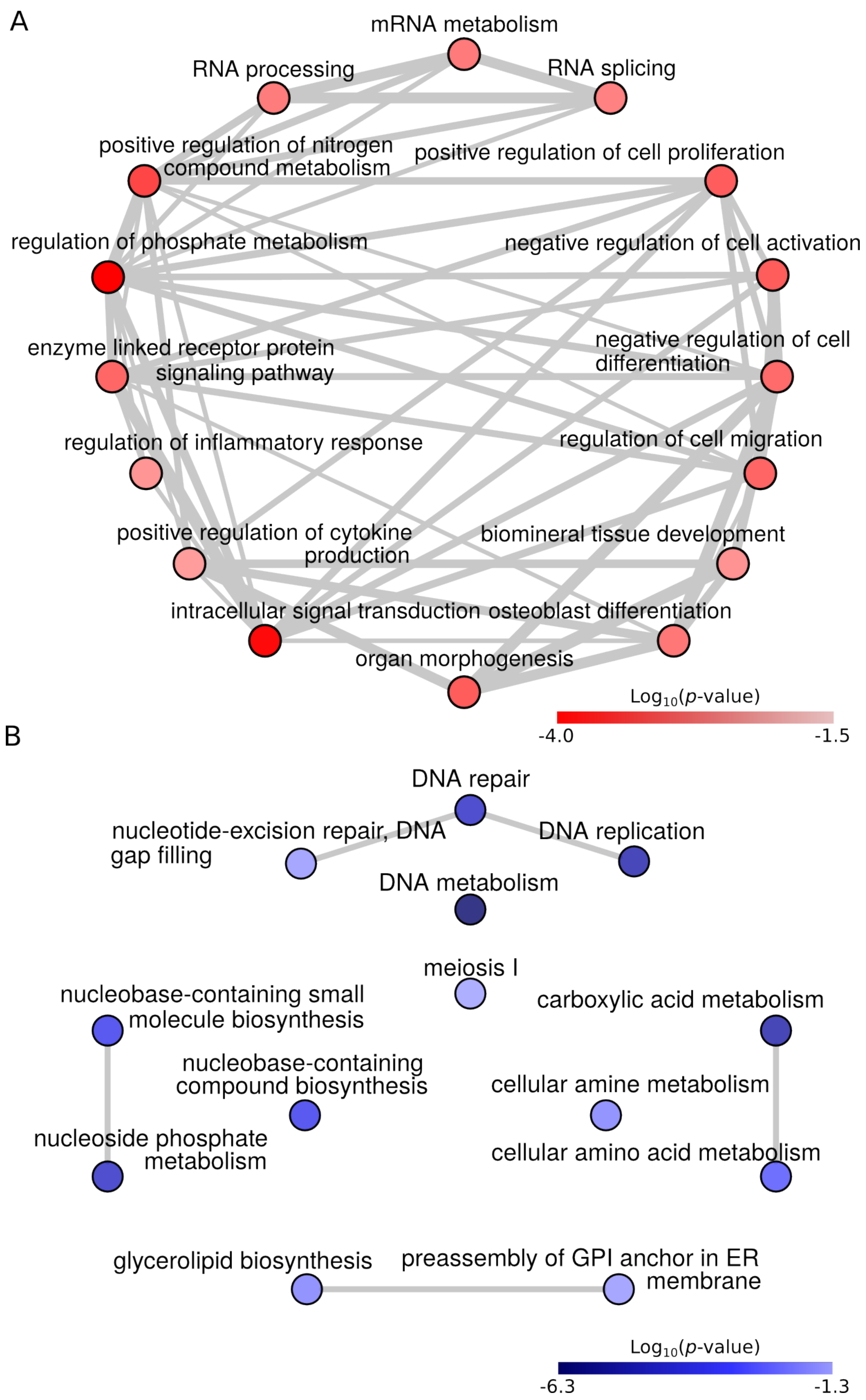


**Figure S9. Gene ontology annotation of differentially expressed genes.** Enriched GO terms are shown for upregulated (**A**) and downregulated (**B**) genes.

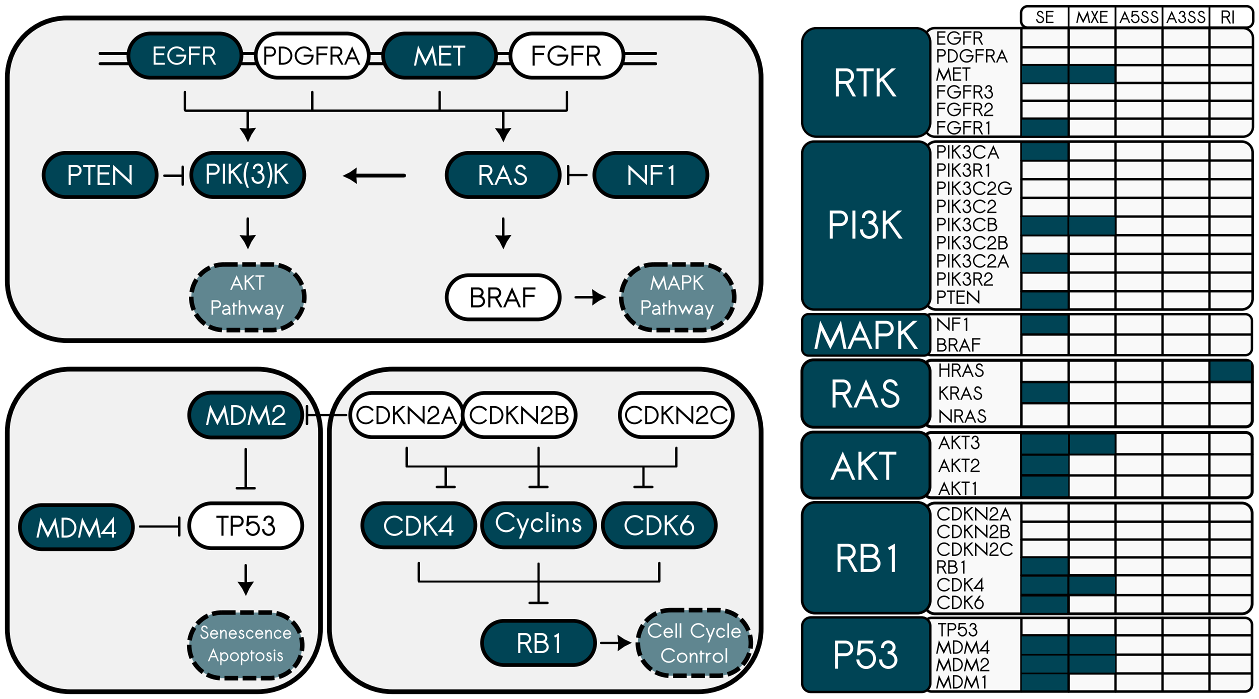


**Figure S10.** **SNRPB knockdown impacts genes/pathways associated with gliomagenesis.** Several genes within critical pathways relevant to GBM origin and development presented splicing alterations upon SNRPB knockdown (highlighted in green). Adapted from [1, 2].

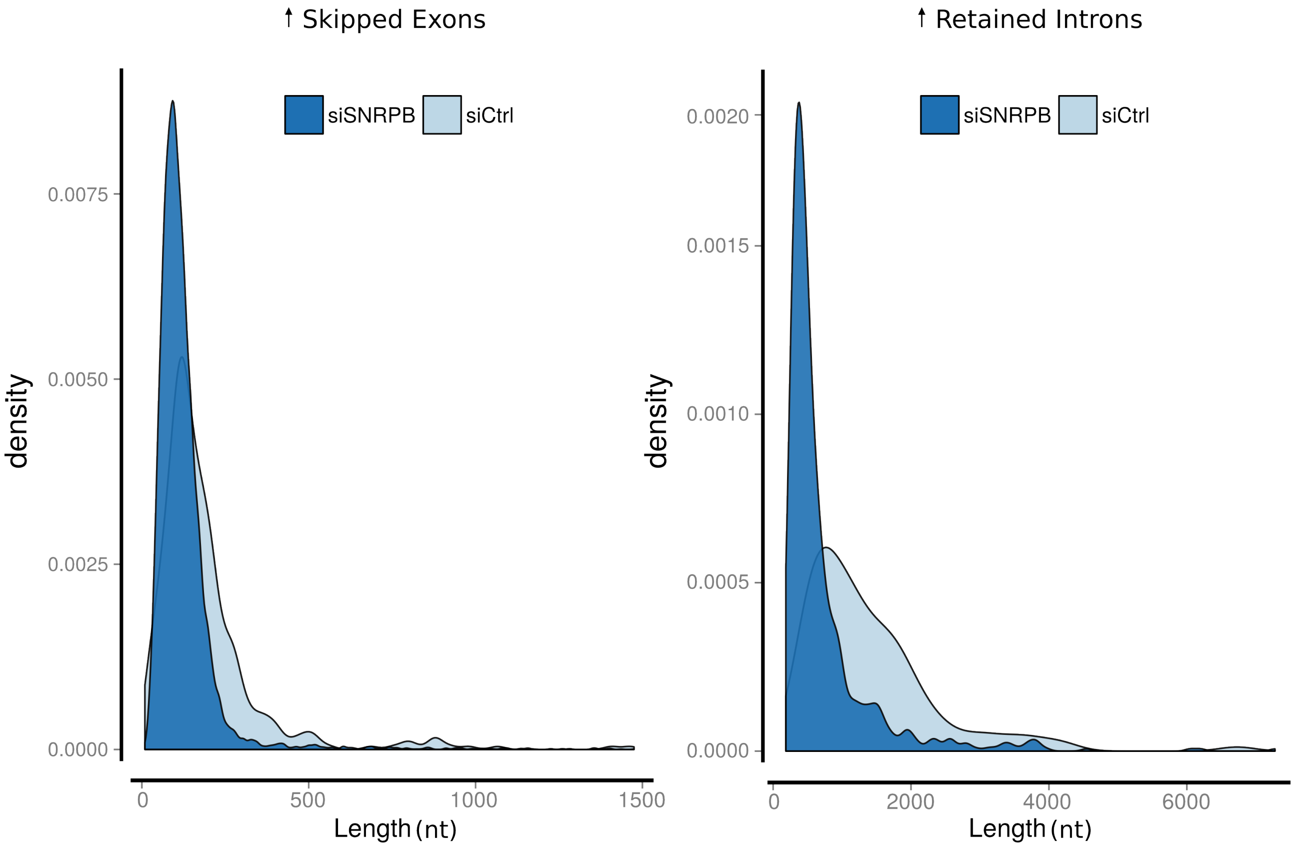


**Figure S11. Length of differentially spliced exons and introns.** Exons with lower inclusion levels upon knockdown are shorter than the ones with lower inclusion levels in control (median knockdown = 106 nt, median control = 148 nt; *p*-value < 2.2e-16; Wilcoxon rank-sum). Introns more retained upon SNRPB knockdown are shorter than the ones more retained in control (median knockdown = 483 nt, median control = 1,144 nt; *p*-value < 2.2e-16; Wilcoxon-rank sum).

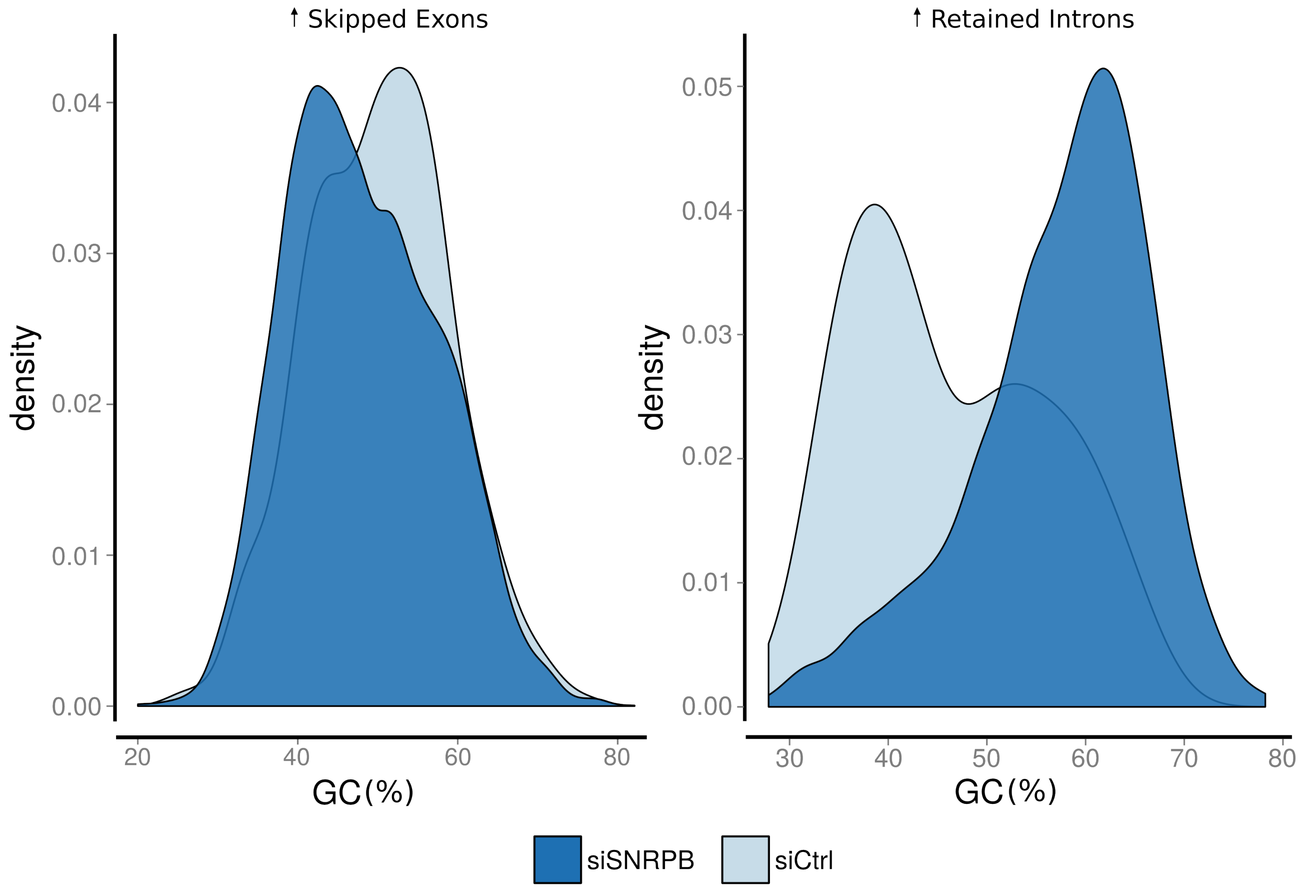


**Figure S12. GC content of differentially spliced exons and introns.** Exons with lower inclusion levels present lower GC% compared to control (median knockdown = 47.25%, median control = 50.57%; *p*-value = 5.936e-07; Wilcoxon rank-sum). Introns more included upon knockdown present higher GC% compared with the ones more retained in control (median knockdown = 59.07%; median control = 43.96%; *p*-value < 2.2e-16; Wilcoxon rank-sum).

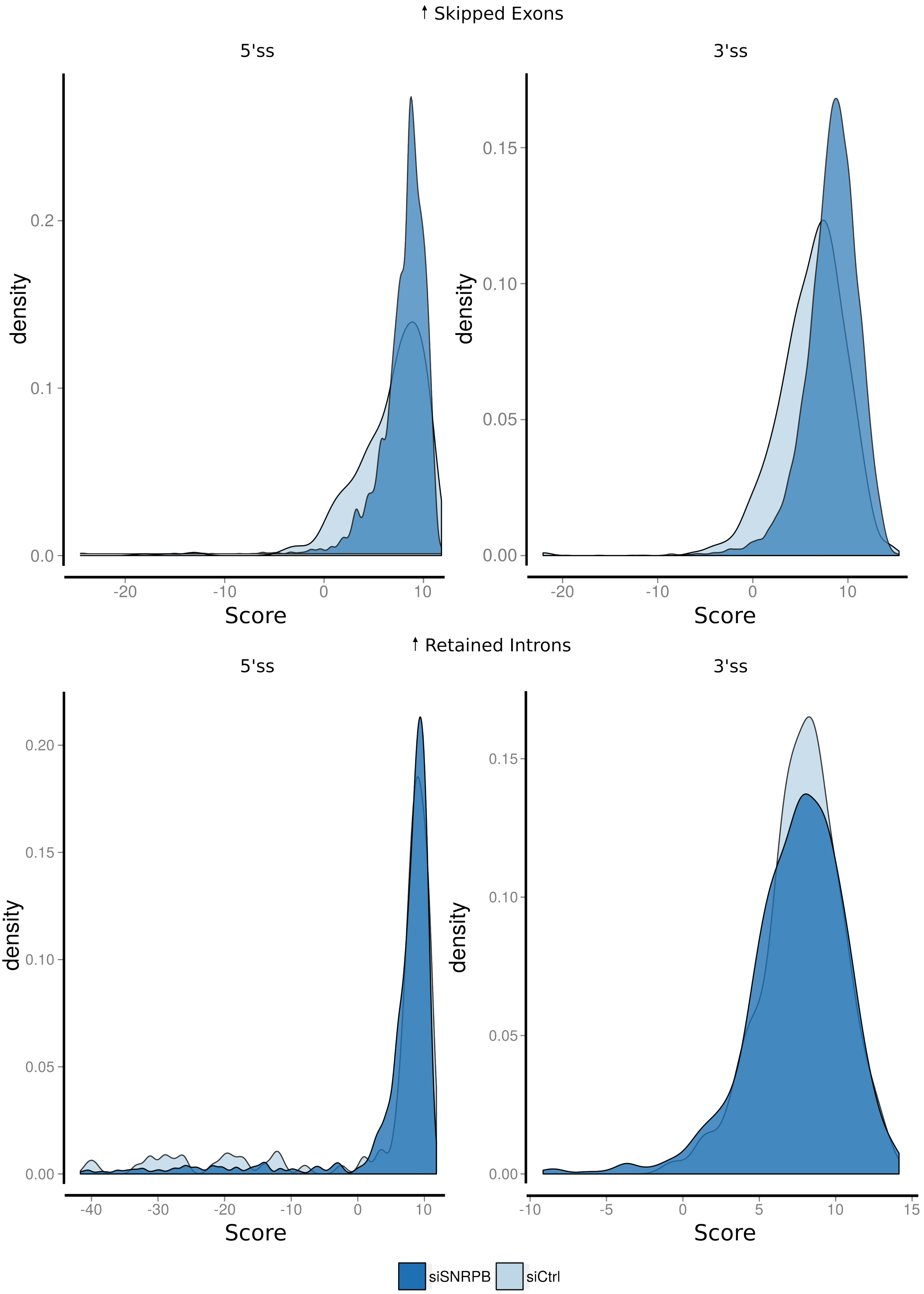


**Figure S13. 3'ss and 5'ss strength of differentially spliced exons/introns.** Exons with lower inclusion levels upon knockdown presented stronger 3'ss and 5'ss (3'ss *p*-value < 2.2e-16; 5'ss *p*-value = 2.092e-14; Wilcoxon rank-sum). No significant difference was observed in introns (3'ss *p*-value = 0.4464; 5'ss *p*-value = 0.9095; Wilcoxon rank-sum).

## References

1. McLendon R, Friedman A, Bigner D, Van Meir EG, Brat DJ, M. Mastrogianakis G, Olson JJ, Mikkelsen T, Lehman N, Aldape K, Alfred Yung WK, Bogler O, VandenBerg S, Berger M, Prados M, Muzny D, Morgan M, Scherer S, Sabo A, Nazareth L, Lewis L, Hall O, Zhu Y, Ren Y, Alvi O, Yao J, Hawes A, Jhangiani S, Fowler G, San Lucas A, et al.: **Comprehensive genomic characterization defines human glioblastoma genes and core pathways**. *Nature* 2008, **455**:1061–1068.

2. Brennan CW, Verhaak RGW, McKenna A, Campos B, Noushmehr H, Salama SR, Zheng S, Chakravarty D, Sanborn JZ, Berman SH, Beroukhim R, Bernard B, Wu C-J, Genovese G, Shmulevich I, Barnholtz-Sloan J, Zou L, Vegesna R, Shukla SA, Ciriello G, Yung WK, Zhang W, Sougnez C, Mikkelsen T, Aldape K, Bigner DD, Van Meir EG, Prados M, Sloan A, Black KL, et al.: **The Somatic Genomic Landscape of Glioblastoma**. *Cell* 2013, **155**:462–477.
